# Amide proton transfer and arterial spin labeling for non-invasive molecular stratification of glioma: a multi-dataset imaging biomarker study

**DOI:** 10.1007/s00234-026-04006-8

**Published:** 2026-04-23

**Authors:** Rajeev Essed, Ivar J. Wamelink, Jan Petr, Joost Kuijer, Alle Meije Wink, Shuncong Wang, Frederik Barkhof, Vera C. Keil

**Affiliations:** 1 Department of Radiology & Nuclear Medicine, Amsterdam UMC Location VUmc, Amsterdam, Netherlands; 2 Cancer Center Amsterdam, Imaging and Biomarkers, Amsterdam, Netherlands; 3 Institute of Radiopharmaceutical Cancer Research, Helmholtz-Zentrum Dresden-Rossendorf, Dresden, Germany; 4 Brain Imaging, Amsterdam Neuroscience, Amsterdam, Netherlands; 5 Department of Radiology, University of Cambridge, Cambridge, UK; 6 Queen Square Institute of Neurology and Centre for Medical Image Computing, University College London, London, UK

**Keywords:** Arterial spin labeling, Amide proton transfer, Chemical exchange saturation transfer, Glioma, IDH mutation, Radiomics

## Abstract

**Purpose:**

To evaluate amide proton transfer chemical exchange saturation transfer magnetization transfer ratio asymmetry (MTR_asym_) and arterial spin labeling cerebral blood flow (CBF), two advanced MRI-based maps, for predicting glioma isocitrate dehydrogenase (IDH) mutation status, 1p/19q codeletion status, and grade using radiomics features; to assess cross-dataset/cross-vendor generalizability; and to examine whether combining MTR_asym_ and CBF features improves prediction.

**Methods:**

This multi-center study included 219 grade 2–4 glioma patients from three datasets (Netherlands/D1: *n* = 48, Siemens, prospective; Russia/D2: *n* = 42, Philips, retrospective; China/D3: *n* = 129, Siemens, retrospective). Descriptive and first-order radiomic features were analyzed from MTR_asym_ (*n* = 219) and CBF maps (*n* = 90, CBF not available in D3), with and without normalization to contralateral regions of interest (ROI). Univariate diagnostic performance was assessed using area under the receiver operating characteristic (AUC) curves, random forest radiomics classifiers were evaluated using 5-fold cross-validation on D1 and subsequently validated externally on D2/D3. Combined MTR_asym_+CBF models were assessed on D1/D2 (*n* = 90).

**Results:**

Tumor-to-contralateral ROI-normalized ratios were comparable across sites (*p* > 0.05). Univariate MTR_asym_ achieved AUCs of 0.94 (1p/19q), 0.87 (grade), and 0.76 (IDH); contralateral ROI-normalized CBF performed comparably for IDH (AUC = 0.77) and 1p/19q (AUC = 0.75), but not grade (AUC = 0.72). Radiomics models achieved AUCs of 0.91 (1p/19q), 0.81 (IDH), and 0.89 (grade) for MTR_asym_; combined MTR_asym_+CBF models significantly improved AUCs of 0.85 (IDH, *p* = 0.007) and 0.91 (grade, *p* = 0.001). External validation revealed MTR_asym_ performed well on D3 (Siemens, same vendor), while normalized CBF generalized better than MTR_asym_ to D2 (Philips).

**Conclusion:**

MTR_asym_ and CBF features show promise for glioma molecular stratification, and multimodal modeling improves predictions. Cross-vendor validation reveals modality-specific generalizability patterns.

**Supplementary Information:**

The online version contains supplementary material available at 10.1007/s00234-026-04006-8.

## Introduction

Gliomas are the most common primary malignant brain tumors and remain a critical neuro-oncological challenge, with glioblastoma (WHO grade 4) carrying a dismal prognosis despite aggressive therapy [1]. Tumor characterization and molecular profiling are vital for prognosis and treatment planning under the current WHO classification [2, 3] and rely on invasive surgical procedures to obtain tissue for analysis. Such procedures carry risks like infection, hemorrhage, and neurological deficit, while not always capturing the molecular heterogeneity of the tumor [4, 5]. Conventional MRI, including gadolinium-based contrast agent (GBCA)-enhanced sequences, is indispensable for initial diagnosis and surgical planning [6]. Still, it exposes patients to the potential risks of repeated contrast agent administration [7, 8].

Advanced MRI techniques like arterial spin labeling (ASL) and amide proton transfer (APT) imaging aim to reduce reliance on more invasive methods requiring GBCA or positron emission tomography (PET)-tracer injection [9]. ASL, a perfusion-weighted imaging (PWI) technique, quantifies cerebral blood flow (CBF) using blood protons as an endogenous tracer [10–13]. APT, on the other hand, is based on chemical exchange saturation transfer (CEST), detecting amide protons resonating at 3.5 parts per million (ppm) downfield from water [14–16]. APT-CEST-derived magnetization transfer ratio asymmetry (MTR_asym_) and ASL-derived CBF maps have previously demonstrated promise in predicting tumor grade, isocitrate dehydrogenase (IDH) mutation status, and 1p/19q codeletion status and have potential as non-GBCA-dependent clinical assessment tools with comparable efficiency [17–22]. However, most APT-CEST and ASL literature lacks external validation datasets and uses varying pulse sequence parameters and post-processing methods, hindering external generalizability [15, 23, 24].

Radiomics offers a framework to overcome these limitations by extracting quantitative features from MRI scans, allowing for the identification of tumor heterogeneity patterns that may correlate with tumor biology [25–27]. While histogram features are criticized for reproducibility issues, they could potentially reduce vendor-specific variability by characterizing voxel distributions rather than absolute intensities, provided appropriate harmonization is applied [28].

We hypothesize that combining perfusion (CBF) and proteomic (MTR_asym_) features within a comprehensive machine learning framework that relies on a larger combined multi-vendor dataset will synergistically improve the non-invasive prediction of histopathological characteristics (IDH, 1p/19q codeletion and WHO grade), as CBF and MTR_asym_ may serve as imaging proxies for distinct yet complementary biological processes, tumor angiogenesis and elevated protein metabolism, respectively [15, 29–31]. As literature on multimodal MTR_asym_+CBF radiomics for glioma mutation prediction is scarce and there is no current research investigating cross-vendor/cross-dataset generalizability, this study has two interrelated aims: first, to evaluate whether the combination of MTR_asym_ and CBF features improves classification of IDH mutation status, 1p/19q codeletion and WHO grade beyond either modality alone; and second, to demonstrate that histogram-based radiomics can yield reproducible and clinically meaningful results when coupled with appropriate cross-site validation and harmonization strategies. Clinically, this approach could support preoperative stratification, biopsy targeting, and follow-up monitoring, potentially offering a feasible addition to routine MRI workflows.

## Materials and methods

### Overview

This study comprised five main analytical components: (i) characterization of MTR_asym_ and CBF signal distributions across three international datasets, including assessment of inter-site and inter-vendor variability; (ii) univariate group comparisons and diagnostic performance evaluation of MTR_asym_ and CBF histogram features for classifying IDH mutation status, 1p/19q codeletion, and WHO grade using ROC analyzes and logistic regressions; (iii) development of Random Forest classifiers using first-order radiomic features from single and combined modalities (MTR_asym_+CBF), validated internally with 5-fold cross-validation over 10 repeats; (iv) assessment of cross-site and cross-vendor generalizability through external validation on two independent datasets (Russia, *n* = 42, Philips; China, *n* = 129, Siemens); and (v) evaluation of classification performance when pooling multi-center data. 

### Patient selection and inclusion

Data acquisition in the Netherlands was approved by the institutional review board, and all patients provided written informed consent. The external validation datasets were approved by their respective institutions, with written informed consent obtained from all patients [32–34].

Participants for the internal dataset were prospectively included between November 2022 and February 2025 meeting the following criteria: (i) pre-operative brain tumor imaging protocol MRI plus APT-CEST, and/or ASL sequences present; (ii) pathological verification of adult diffuse glioma diagnosis (according to the WHO CNS criteria (CNS5 2021) [3] present); (iii) availability of molecular/histopathological data, including IDH mutation status, 1p/19q codeletion status, and tumor grade [35].

Exclusion criteria were: (i) age < 18 years; (ii) diagnosis not clearly fitting adult-type diffuse glioma; (iii) corrupted MTR_asym_ or ASL CBF maps. 

### MRI acquisition and pre-processing

#### Internal dataset (Netherlands, D1)

Scanning was performed at 3T (Vida, Siemens Healthineers, Erlangen, Germany) with a 20-channel head coil. The protocol included 3D T1-weighted MPRAGE (TR/TE/TI = 2300/2.32/900 ms, flip angle = 8°, resolution = 0.9 mm isotropic, FoV = 240 mm, 192 sagittal slices, GRAPPA = 2), T2-weighted TSE (TR/TE = 4100/74 ms, flip angle = 150°, resolution = 0.4 × 0.4 × 5.0 mm³, FoV = 220 mm, 28 transversal slices), 3D FLAIR (TR/TE/TI = 5000/388/1650 ms, T2 prep = 125 ms, resolution = 0.5 × 0.5 × 0.9 mm³, FoV = 230 mm, 176 sagittal slices, GRAPPA = 2), post-contrast 3D T1-weighted MPRAGE (identical parameters to pre-contrast), multi-post labeling delay (PLD) pseudocontinuous ASL (pCASL) sequences, and a 3D TSE SPACE-CEST sequence. Detailed ASL and APT-CEST sequence parameters can be found in Tables S1 and S2. APT-CEST quantitative maps were calculated using the magnetization transfer ratio asymmetry (MTR_asym_) at 3.5 ppm, defined as:

$$\:AP{T}_{CEST}\:=MT{R}_{asym}\left(3.5ppm\right)\frac{S(-3.5ppm)-S(+3.5ppm)}{S0}$$

Where S represents the signal intensity at the specified offset frequency, and S0 is the reference signal with a specific saturation. CBF maps were quantified using ExploreASL [36].

#### External dataset 1 (Russia, D2)

Imaging was conducted on a 3T MRI system (Ingenia, Philips Healthcare, Best, The Netherlands) utilizing a 16-channel head coil (32). The acquisition protocol comprised 3D T1-weighted TFE (TR/TE = 6.63/3.01 ms, flip angle = 8°, resolution = 1 mm isotropic, matrix = 240 × 240 × 192, SENSE = 2.2), 3D T2-weighted TSE (TR/TE = 3500/300 ms, resolution = 1 mm isotropic, matrix = 252 × 252 × 180, SENSE = 2.4 × 2.1, fat saturation), 3D FLAIR (TR/TE/TI = 4800/340/1650 ms, resolution = 1.2 mm isotropic, matrix = 228 × 228 × 140, SENSE = 3 × 1.7, fat saturation), T1-weighted post-contrast (identical parameters to pre-contrast T1), SWI, DWI, ASL, and APT-CEST sequences. Detailed ASL and APT-CEST sequence parameters can be found in Tables S1 and S2. MTR_asym_ maps were calculated by the MRI scanner. Participants were adults histologically diagnosed with diffuse brain gliomas according to the WHO CNS criteria (CNS5 2021), and collected at Federal Neurosurgical Center, Novosibirsk, Russia, in 2023.

#### External dataset 2 (China, D3)

Imaging of the D3 [34] was conducted on a 3T MRI system (Prisma, Siemens Healthcare, Erlangen, Germany), which included T1-weighted, T2-weighted, FLAIR, and T1-weighted post-contrast and APT-CEST imaging. Detailed APT-CEST sequence parameters are provided in Table S1, and quantitative maps were calculated in the same manner as for D1. Participants were adults with diagnosed diffuse brain gliomas according to the WHO CNS criteria (CNS5 2021), and collected at Tiantan Hospital, Beijing, China, from December 2020 to August 2022.

### Registration and segmentation

Tumors were segmented automatically using the picture nnUNet package (version 0.6.1) [37] on the structural MRI sequences and subsequently manually adjusted if necessary by two researchers under the supervision of a neuroradiologist using ITK-SNAP (version 3.8.0) on D1 and D2. All researchers were blinded, and disagreements were resolved through consensus discussions. To avoid uncertainty regarding APT/ASL-based regions of interest (ROI), three distinct tumor compartments were delineated based on 3D structural MRI sequences: non-enhancing tumor/edema (label 1, based on FLAIR hyperintensity), necrotic tumor core (label 2, based on T1 hypointensity inside T1-post contrast tumor borders), and enhancing tumor (label 3, based on T1-post contrast enhancing lesion parts).

**Fig. 1:**
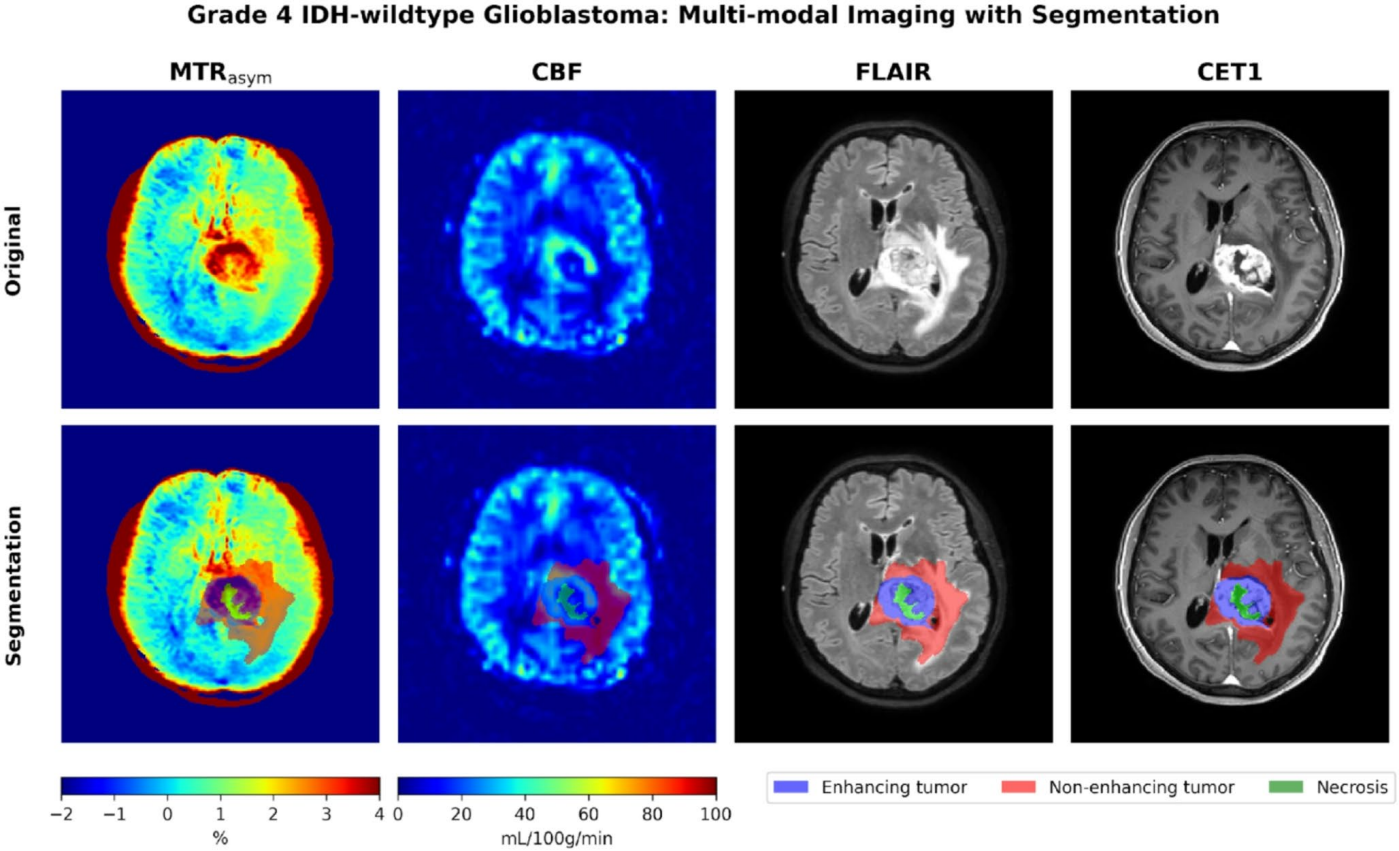
Magnetization transfer ratio asymmetry (MTR_asym_), cerebral blood flow (CBF), Fluid-Attenuated Inversion Recovery (FLAIR), and post-contrast T1 (CET1) scans of a grade 4 IDH-wildtype glioma case with segmentation labels for enhancing tumor (blue), non-enhancing tumor (red) and necrosis (green)

The tumor ROI was defined by the enhancing tumor region (label 3) in the case of an enhancing tumor. In the absence of tumor enhancement, the non‑enhancing tumor components (label 1) were selected as the tumor ROI. Necrotic regions (label 2) were removed from the segmentation due to lack of fluid suppression in the APT-CEST scans in D1 and D3. A visual representation of the ROI selection is shown in Fig. 1 in an IDH-wildtype case in D2.

For D1, APT-CEST and ASL sequences were co-registered to the corresponding 3D T1-weighted images. In contrast, in D2, structural and ASL-sequences were co-registered to APT-CEST images to minimize interpolation artifacts resulting from slice thickness variations. Registrations were performed using a rigid body transformation in FLIRT (FSL version 5.0.9). Image pre- and post-processing for D3 is described by Wu et al. [34].

### Feature extraction

Mean MTRasym and CBF values were extracted from five regions: whole brain, white matter (WM), grey matter (GM), the tumor ROI, and a homologous contralateral ROI. The contralateral ROI was created by mirroring the lesion mask to the opposite hemisphere and restricting analysis to normal-appearing white and grey matter voxels, thereby excluding cerebrospinal fluid, edema, and regions affected by midline shift. Additionally, for D1 and D2, first-order histogram features were extracted to characterize the distribution of MTR_asym_ and CBF values within the tumor ROI to focus on fundamental intensity-based differences across datasets. Using the open-source PyRadiomics library (version 3.0 [38]), we extracted 18 first-order features, listed in the Supplementary material. We restricted our analysis to first-order features as these are generally more reproducible across acquisition protocols and scanner platforms than higher-order texture features, which are highly sensitive to voxel size, resolution, and reconstruction methods [39]. All features were computed separately for the raw MTR_asym_ and CBF maps. Features from D3 had already been computed, and the source data could not be obtained [34].

### Statistical analysis

#### Dataset comparisons

D1 and D2 had MTR_asym_ and CBF maps available alongside contralateral ROI reference tissue, while D3 only contained pre-computed MTR_asym_ features. For D1 and D2, both absolute tumor values and contralateral ROI-normalized tumor values (calculated as the tumor-to-contralateral ROI ratio) were computed for MTR_asym_ and CBF.

Mean MTR_asym_ and CBF signal intensities were compared between datasets and vendors using pairwise Mann-Whitney U tests to identify differences. The comparisons included: mean absolute tumor value, mean contralateral ROI value, and contralateral ROI-normalized tumor value for MTR_asym_ and CBF for datasets where available. Additionally, mean absolute tumor values were compared between enhancing versus non-enhancing tumors within D1 and D2.

#### Group comparisons and diagnostic performance

Using the D1 dataset, univariate diagnostic performance was assessed for three binary classification tasks: IDH status (mutant versus wildtype), 1p/19q codeletion status (codeleted versus non-codeleted), and WHO grade (low (2) versus high grade (3–4)). Three histogram features were selected for analysis: mean signal intensity, 90th percentile signal intensity, and maximum signal intensity. These metrics were chosen to capture central tendency and the upper tail of the signal distribution within tumor regions, as elevated signals in the most metabolically active tumor subregions may provide complementary diagnostic information. Absolute tumor values and contralateral ROI-normalized values were analyzed for MTR_asym_ and CBF.

To investigate signal differences across histologic subtypes in D1, we performed two separate analyses using different tumor segmentation approaches: first, using our previously established labeling strategy, and second, using a combined mask of enhancing tumor and FLAIR hyperintense tumor (label 3 + 1). The combined mask approach reduces bias inherent to using enhancing tumor regions alone, as enhancement may already correlate with higher grade and more aggressive disease. Mean signal intensities were extracted from tumor regions and contralateral normal-appearing white matter (NAWM) to calculate tumor-to-NAWM ratios.

Group comparisons between molecular subgroups in D1 were performed using Mann-Whitney U tests for two-group comparisons and Kruskal-Wallis tests for three-group comparisons, with rank-biserial correlation (r) and matched-pairs rank-biserial correlation effect sizes respectively. Multiple-comparison correction was applied using the Benjamini-Hochberg false discovery rate (FDR) method across all histogram features and molecular predictions and applies to all reported p-values. These comparisons are used to justify the selection of features (e.g. contralateral ROI normalized or not) for the diagnostic performance assessments. Diagnostic performance was evaluated using receiver operating characteristic (ROC) curve analysis. Area under the ROC curve (AUC) with 95% confidence intervals was calculated using 2000 bootstrap iterations. To assess whether combining imaging modalities improved classification, logistic regression was used to integrate MTR_asym_ and CBF features, and DeLong tests were employed to compare AUCs between single-modality and combined models. 

#### Radiomics and machine learning

Machine learning classification was performed to evaluate the predictive value of radiomic features for the same three binary classification tasks (IDH status, 1p/19q codeletion status, and WHO grade). A Random Forest classifier was used to model non-linear relationships between radiomic features and molecular status and its tree-based ensemble design. The classifier was implemented using scikit-learn (version 1.5.2) [40] with the following parameters: 100 estimators, maximum depth of 5, minimum samples per split of 10, and minimum samples per leaf of 5. These conservative hyperparameters were chosen to limit model complexity and reduce the risk of overfitting, given the relatively small dataset sizes. The feature set consisted of all 18 first-order radiomic statistics extracted from tumor regions.

Model performance was first established on the D1 dataset using 10 repetitions of 5-fold stratified cross-validation, yielding 50 AUC values per model configuration. Three feature configurations were evaluated: MTR_asym_-only features, CBF-only features (tested with both raw and Contralateral ROI-normalized versions), and combined MTR_asym_ and CBF features. The mean AUC and 95% confidence intervals were calculated from the distribution of the 50 AUC values using the t-distribution. Given that dataset-specific univariate and/or radiomics results have been previously published for D2 and D3, our study focused on combined and cross-site analyzes.

To assess generalizability across institutions and vendors, models trained on D1 were subsequently applied to D2 and D3 as external validation datasets. Performance was evaluated after Z-score normalization, where the feature-wise mean and standard deviation were estimated exclusively from the D1 training set and applied without re-estimation to the external cohort using the scikit-learn (version 1.5.2) StandardScaler. Given the absence of CBF data in D3, this dataset was used only to validate MTR_asym_’s generalizability.

Finally, to evaluate whether pooling data from multiple centers could improve classification performance, datasets were combined and evaluated using 5-fold stratified cross-validation. Pooling with Z-score harmonization to the D1 reference distribution was used. Four dataset combinations were tested to account for differences in data availability: MTR_asym_ features across all three datasets, MTR_asym_ features across D1 and D2 only, CBF features across D1 and D2, and combined MTR_asym_ and CBF features across D1 and D2.

To assess whether combined MTR_asym_ and CBF features provided synergistic improvement over single-modality models, paired t-tests were performed on the AUC values obtained from repeated cross-validation on the D1 internal diagnostic performance evaluation. Since both models were evaluated on identical fold partitions across all 10 repetitions, the resulting 50 AUC values were paired, allowing for a direct comparison that accounts for fold-to-fold variability. For external and pooled multi-dataset evaluations, all AUC comparisons between the combined and unimodal models were performed using the DeLong test. ΔAUC is the difference between the multimodal model and the best performing unimodal model. Statistical significance was defined as *p* < 0.05.

## Results

### Dataset comparisons

Patient demographics are provided in Table 1 and an inclusion flow diagram can be found in Fig. S1. Table 2 presents mean values for MTR_asym_ and CBF across the whole brain, contralateral ROI, and tumor ROI, stratified by tumor type and grade in D1. Supplementary Table S3 provides absolute and normalized histogram features (mean, 90th percentile, and maximum values) for different molecular subtypes based on IDH and 1p/19q codeletion status in D1.

**Table 1:** Demographics per dataset

Number of patients	Internal (NL), D1	External 1 (RU), D2	External 2 (CN), D3
	48	42	129
Age (years)	56.6 ± 15.0	53.5 ± 14.3	54.64 ± 12.86
Sex (F: M)	21:27	23:19	60:69
IDH state (wildtype: mutated)	27:21	15:27	88:41
1p/19q state (codeleted: non-codeleted)	7:41	8:34	29:100
WHO grade distribution (2/3/4)	13/5/30	5/13/24	77/52*

Abbreviations: *1p/19q *Chromosome 1p and chromosome 19q, *CN* China, *D1* Dataset 1, *D2* Dataset 2, *D3* Dataset 3,* F* Female,* IDH* Isocitrate dehydrogenase, *M* Male, *NL* Netherlands, *RU* Russia, *WHO* World Health Organization

* Only binary data available: high (3/4) versus low (2)

**Table 2:** Mean MTRasym and CBF values for the different tumor types and tumor grades in D1

Group	*N*	Modality	Tumor ROI	Contralateral ROI	WM	GM	Whole brain
IDH-mutant 1p/19q-codel oligodendroglioma	7	MTR_asym_	1.53 ± 0.42	0.89 ± 0.64	0.62 ± 1.45	0.86 ± 1.05	0.80 ± 1.15
IDH-mutant 1p/19q-codel oligodendroglioma	7	CBF	30.66 ± 19.29	37.20 ± 33.07	24.46 ± 14.89	50.64 ± 14.57	38.80 ± 26.08
IDH-mutant astrocytoma	14	MTR_asym_	1.52 ± 0.58	0.83 ± 0.61	0.48 ± 1.50	0.85 ± 1.05	0.79 ± 1.14
IDH-mutant astrocytoma	14	CBF	32.91 ± 24.29	34.50 ± 28.49	24.77 ± 14.42	50.51 ± 14.79	40.36 ± 23.29
IDH-wildtype glioblastoma	27	MTR_asym_	2.15 ± 0.84	1.03 ± 0.72	0.56 ± 1.48	0.87 ± 1.10	0.80 ± 1.20
IDH-wildtype glioblastoma	27	CBF	43.85 ± 27.14	38.34 ± 26.33	26.07 ± 17.76	51.67 ± 14.81	37.92 ± 22.58
Grade 2	13	MTR_asym_	1.35 ± 0.51	0.96 ± 0.65	0.57 ± 1.49	0.89 ± 1.05	0.82 ± 1.15
Grade 2	13	CBF	29.20 ± 22.21	33.39 ± 26.25	25.21 ± 15.24	49.71 ± 14.39	39.25 ± 24.47
Grade 3	5	MTR_asym_	1.34 ± 0.44	0.88 ± 0.50	0.41 ± 1.40	0.81 ± 1.02	0.72 ± 1.12
Grade 3	5	CBF	47.03 ± 22.90	47.40 ± 33.87	30.16 ± 17.91	54.41 ± 15.21	41.02 ± 25.76
Grade 4	30	MTR_asym_	2.26 ± 0.85	0.99 ± 0.73	0.56 ± 1.49	0.87 ± 1.10	0.80 ± 1.20
Grade 4	30	CBF	42.58 ± 27.18	37.30 ± 27.50	24.91 ± 17.01	51.40 ± 14.86	37.93 ± 22.30

Abbreviations:*1p/19q *Chromosome 1p and chromosome 19q, *APT* Amide Proton Transfer, *ASL *Arterial Spin Labeling, *CBF *Cerebral Blood Flow, *D1 *Dataset 1 from the Netherlands, *IDH *Isocitrate dehydrogenase, *MTR*_asym_ Magnetization Transfer Ratio asymmetry, *N *Number of patients, *NAWM *Normal-Appearing White Matter, *ROI *Region of Interest, *SD *Standard Deviation

**Fig. 2:**
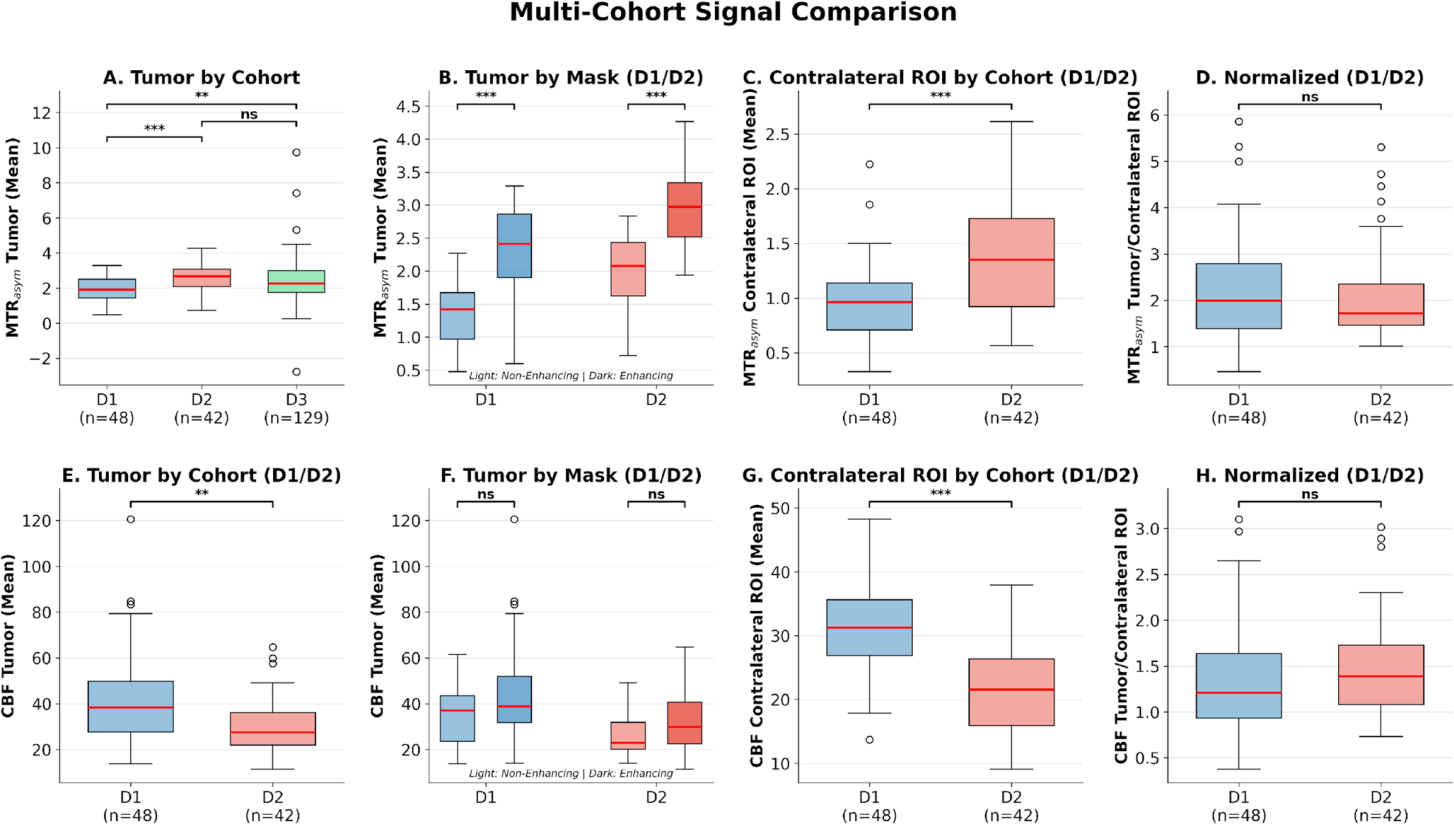
Box plots comparing magnetization transfer ratio asymmetry (MTR_asym_) at 3.5 ppm (top row, A–D) and cerebral blood flow (CBF; bottom row, E–H) across datasets and tumor regions. (**A**) MTR_asym_ tumor signal across the D1, D2, and D3 datasets. (**B**) MTR_asym_ tumor signal in non-enhancing versus enhancing (*n* = 57) tumor regions. (**C**) MTR_asym_ in normal-appearing white matter (NAWM) between the D1 and D2 datasets. (**D**) Normalized MTR_asym_ (tumor/NAWM ratio) between datasets. (**E**) CBF tumor signal between the D1 and D2 datasets. (**F**) CBF tumor signal in non-enhancing versus enhancing tumor regions. (**G**) CBF in NAWM between datasets. (**H**) Normalized CBF (tumor/NAWM ratio) between datasets. ODG = oligodendroglioma; GBM = glioblastoma. Statistical significance (corrected for multiple comparisons): *p < 0.05, **p < 0.01, ***p < 0.001; ns = not significant

Significant inter-dataset/vendor differences were observed in baseline signal intensities: mean tumor MTR_asym_ was significantly lower in D1 (1.94%±0.75%) compared to both D2 (2.61%±0.81%; *p* < 0.001; *r* = 0.44) and D3 (2.42%±1.25%; *p* = 0.007; *r* = 0.26). Mean contralateral ROI MTR_asym_ was also lower in D1 versus D2 (0.97%±0.58% vs. 1.38%±0.56%; *p* < 0.001; *r* = 0.45). Mean CBF values showed the opposite pattern, with D1 demonstrating significantly higher mean tumor CBF (41.97 ± 20.91 vs. 30.09 ± 12.55 ml/100 g/min; *p* = 0.002; *r* = − 0.38) and mean contralateral ROI CBF (31.35 ± 14.41 vs. 21.43 ± 8.44 ml/100 g/min; *p* < 0.001; *r* = − 0.67) compared to D2. However contralateral ROI-normalized ratios were comparable (*p* > 0.05) between datasets (MTR_asym_ normalized: D1 2.25 ± 3.03, D2 2.13 ± 1.42; CBF normalized: D1 1.36 ± 0.63, D2 1.47 ± 0.60). Detailed descriptive statistical comparisons across multiple datasets are shown in Fig. 2.

#### Group comparisons

**Fig. 3:**
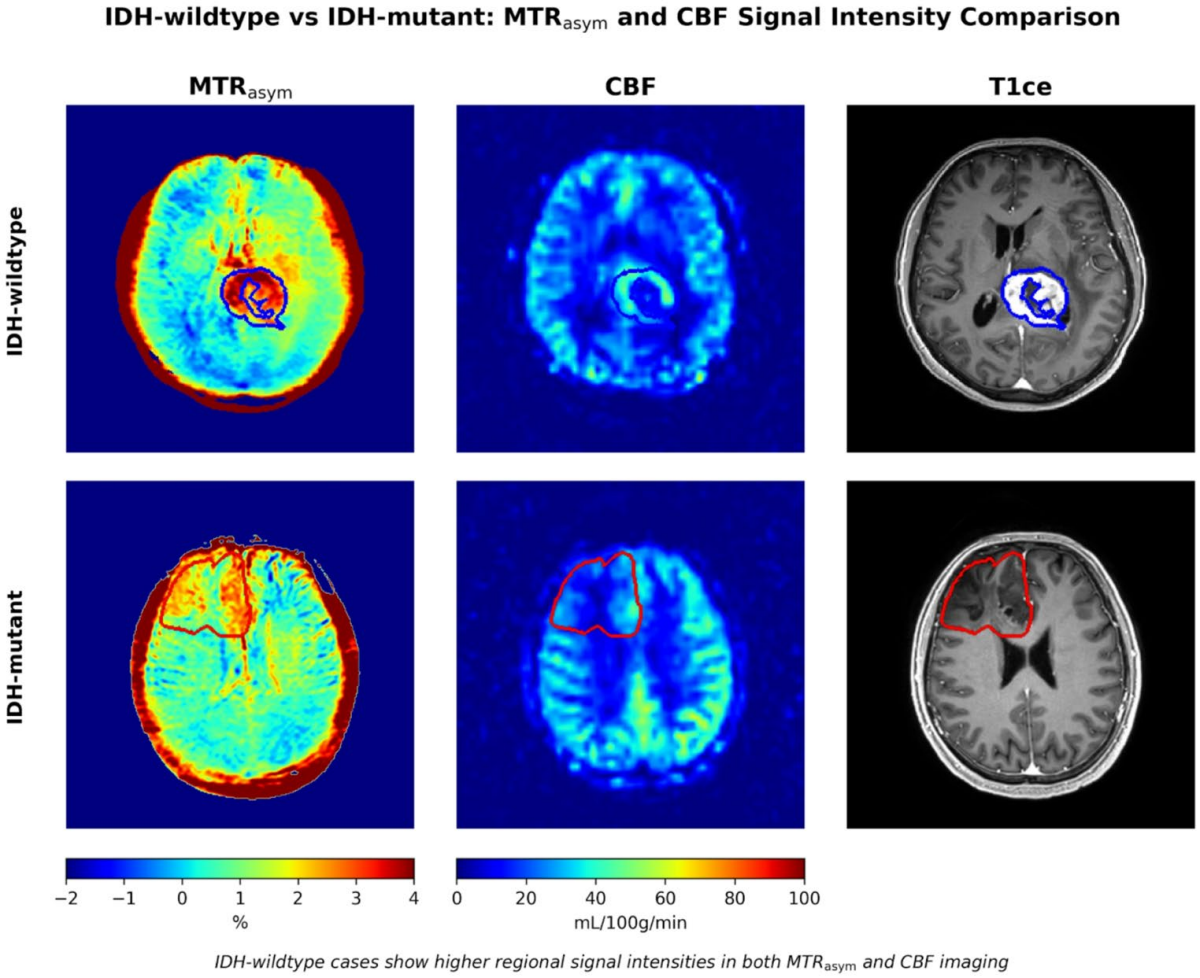
Example of enhancing IDH-wildtype (top row) versus non-enhancing IDH-mutant cases (bottom row) with raw signal intensity in magnetization transfer ratio asymmetry (MTR_asym_) maps and cerebral blood flow (CBF) maps showing higher regional signal intensities for both MTR_asym_ and CBF in the IDH-wildtype case

FDR-corrected group comparisons revealed that MTR_asym_ features were significantly associated with molecular subtypes, whereas CBF features showed more limited associations. For 1p/19q codeletion prediction, only MTR_asym_ maximum was significantly higher in non-codeleted than codeleted tumor patients after correction for multiple comparisons (*p* < 0.001, *r* = − 0.89). For IDH mutation status, MTR_asym_ 90th percentile (*p* = 0.017, *r* = 0.52), mean (*p* = 0.029, *r* = 0.47), and normalized CBF 90th percentile (*p* = 0.017, *r* = 0.54) and mean (*p* = 0.029, *r* = 0.45) were significantly higher in IDH-wildtype compared to IDH-mutant tumors (see Fig. 3 for illustration). Pairwise grade comparisons showed the biggest differences between grade 2 and grade 4 tumors, with MTR_asym_ 90th percentile (*p* = 0.002, *r* = 0.71), mean (*p* = 0.002, *r* = 0.69), and maximum (*p* = 0.012, *r* = 0.53) all significantly higher in grade 4, and normalized CBF 90th percentile (*p* = 0.008, *r* = 0.57) and mean (*p* = 0.023, *r* = 0.48) were also significantly higher in grade 4 compared to grade 2 tumors. Grade 4 tumors showed significantly higher MTR_asym_ 90th percentile (*p* = 0.046, *r* = 0.77) compared to grade 3 tumors, while no features distinguished grade 2 from grade 3 tumors.

In the enhancing tumor mask analysis, glioblastomas exhibited significantly higher mean MTR_asym_ tumor signal than both oligodendrogliomas (*p* = 0.010, *r* = 0.63) and astrocytomas (*p* = 0.003, *r* = − 0.58), with MTR_asym_ significantly elevated in tumors versus contralateral ROIs across all three types (oligodendrogliomas: *p* = 0.016, *r* = 1.0; glioblastomas: *p* < 0.001, *r* = 1.0; astrocytomas: *p* = 0.007, *r* = 0.79). For CBF, GBM showed significantly higher tumor-to-contralateral ratios than oligodendrogliomas (*p* = 0.048, *r* = 0.49) and elevated perfusion within tumors versus contralateral tissue (*p* < 0.001, *r* = 0.72). When expanding the mask to include FLAIR hyperintense tumor, glioblastomas maintained significantly higher MTR_asym_ than oligodendrogliomas (*p* = 0.008, *r* = 0.64) and astrocytomas (*p* = 0.014, *r* = − 0.49), though the paired tumor-to-contralateral elevation remained significant only for glioblastomas (*p* < 0.001, *r* = 1.0) and astrocytomas (*p* = 0.027, *r* = 0.69), losing significance for oligodendrogliomas (*p* = 0.078). Notably, with this expanded mask, CBF showed no significant between-group differences, though glioblastomas maintained elevated tumor perfusion versus contralateral tissue (*p* = 0.021, *r* = 0.50). See supplementary Fig. S2a and b for full tumor type comparisons.

Diagnostic p

#### Diagnostic performance

**Fig. 4:**
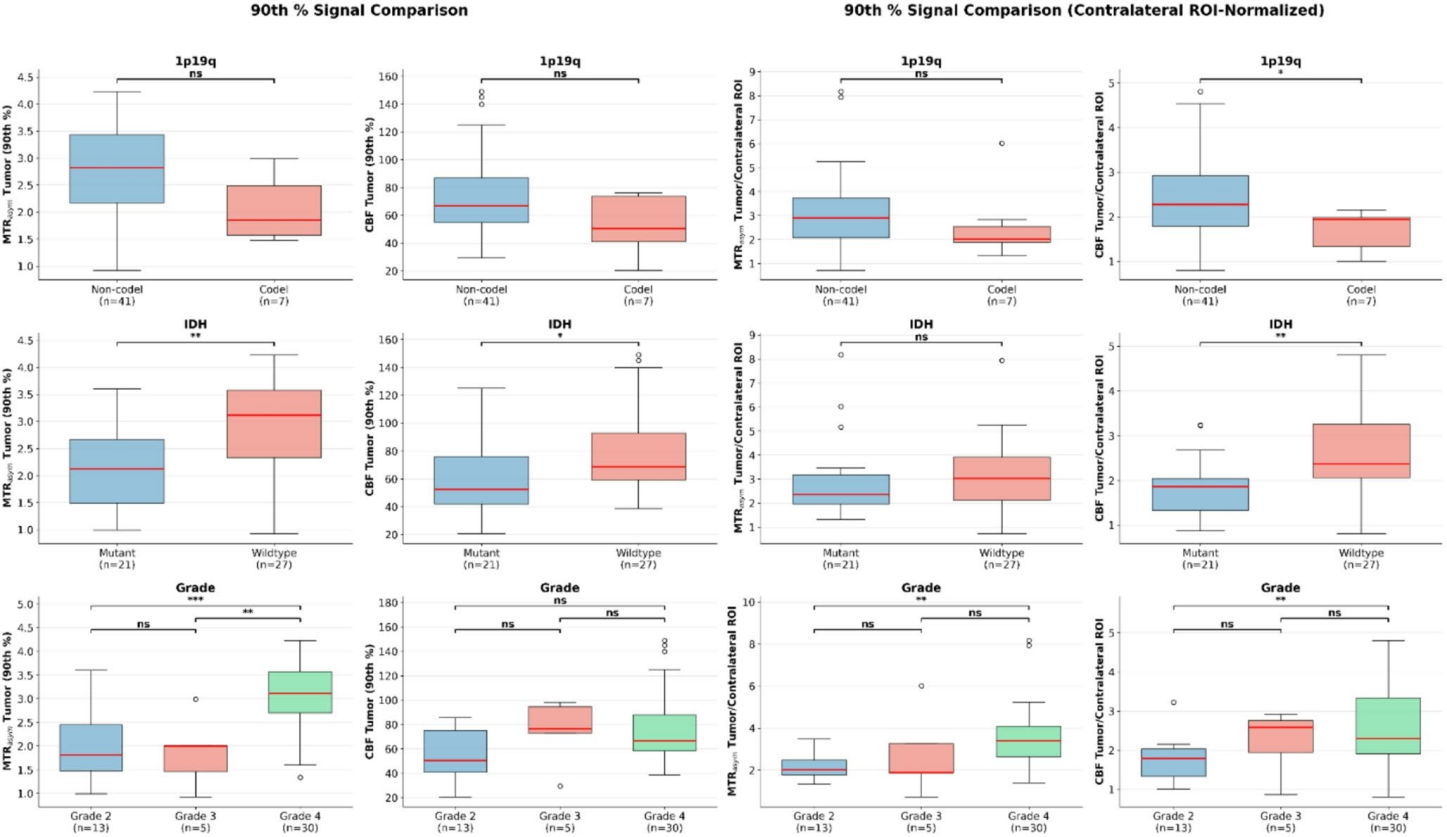
Boxplots comparing raw (left 2 × 3) and contralateral region-of-interest (ROI)-normalized (right 2 × 3) 90^th^ percentile MTR_asym_ and cerebral blood flow (CBF) tumor values between groups for 1p/19q codeletion status (row 1), IDH status (row 2) and grade (row 3) in dataset D1. Statistical significance (corrected for multiple comparisons): *p < 0.05, **p < 0.01, ***p < 0.001; ns = not significant

**Fig. 5:**
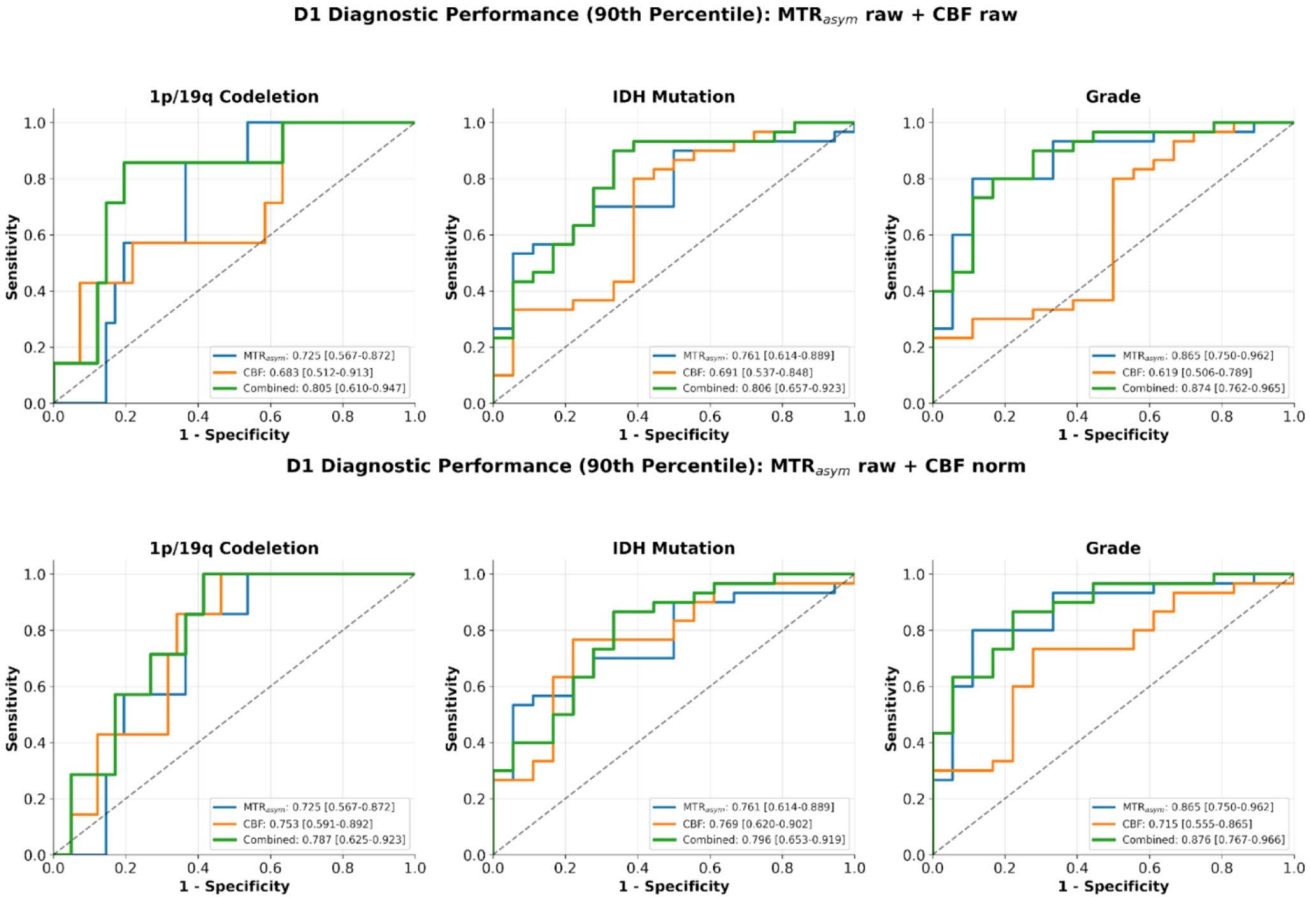
Area under the receiver operating characteristics curves (AUROC) for cerebral blood flow (CBF), magnetization transfer ratio asymmetry (MTR_asym_) and combined (MTR_asym_+CBF) in predicting IDH status, 1p/19q codeletion status, and grade (high versus low) using raw (top row) and normalized by normal appearing white matter (NAWM) (bottom row) 90^th^ percentile tumor values in dataset D1

Discriminative diagnostic performance mirrored these findings. For grade prediction, MTR_asym_ 90th percentile showed strong performance (AUC = 0.87; 95% CI: 0.75–0.96), while normalized CBF 90th percentile achieved moderate discrimination (AUC = 0.72; 95% CI: 0.56–0.87). For IDH prediction, MTR_asym_ and normalized CBF 90th percentile showed comparable moderate performance (AUC = 0.76; 95% CI: 0.61–0.89 and AUC = 0.77; 95% CI: 0.62–0.90, respectively). For 1p/19q codeletion, normalized CBF 90th percentile (AUC = 0.75; 95% CI: 0.59–0.89) slightly outperformed MTR_asym_ (AUC = 0.73; 95% CI: 0.57–0.87). Combining MTR_asym_ and CBF 90th percentile features improved performance for grade (AUC = 0.88; 95% CI: 0.77–0.97), IDH (AUC = 0.80; 95% CI: 0.65–0.92), and 1p/19q prediction (AUC = 0.79; 95% CI: 0.63–0.92), however, DeLong tests were not significant for any comparison. Comparisons of signal intensities between groups and diagnostic performances can be found in Figs. 4 and 5, S3 and S4.

### Radiomics and machine learning

#### Dutch dataset evaluation

**Fig. 6:**
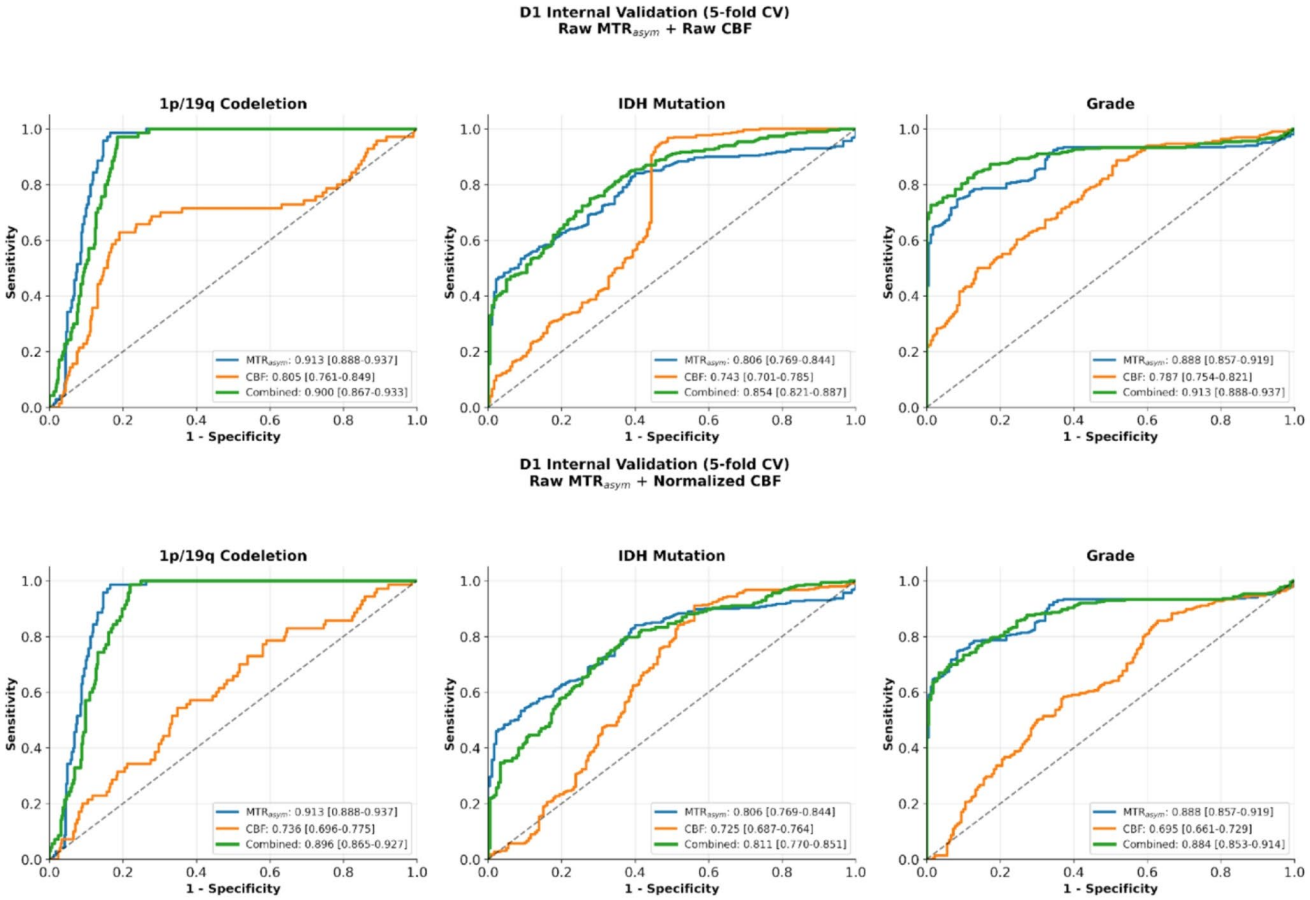
Area under the receiver operating characteristics curves (AUROC) for cerebral blood flow (CBF), magnetization transfer ratio asymmetry (MTR_asym_) and combined (MTR_asym_+CBF) in predicting IDH status, 1p/19q codeletion status, and grade (high versus low) using random forest radiomics models with 5-fold internal cross validation (CV) in dataset D1

The predictive performance of MTR_asym_, CBF, and the combined model for molecular and histological classification in the D1 (*n* = 48) can be found in Fig. 6. Multimodal modeling yielded significant improvements in AUC in IDH (*p* = 0.007, ΔAUC = 0.05) and grade (*p* = 0.001, ΔAUC = 0.03) classification compared to the best single modality. Table S4 provides a side-by-side comparison between the diagnostic performance of the best-performing univariate feature compared to radiomics models.

#### External validation

**Fig. 7:**
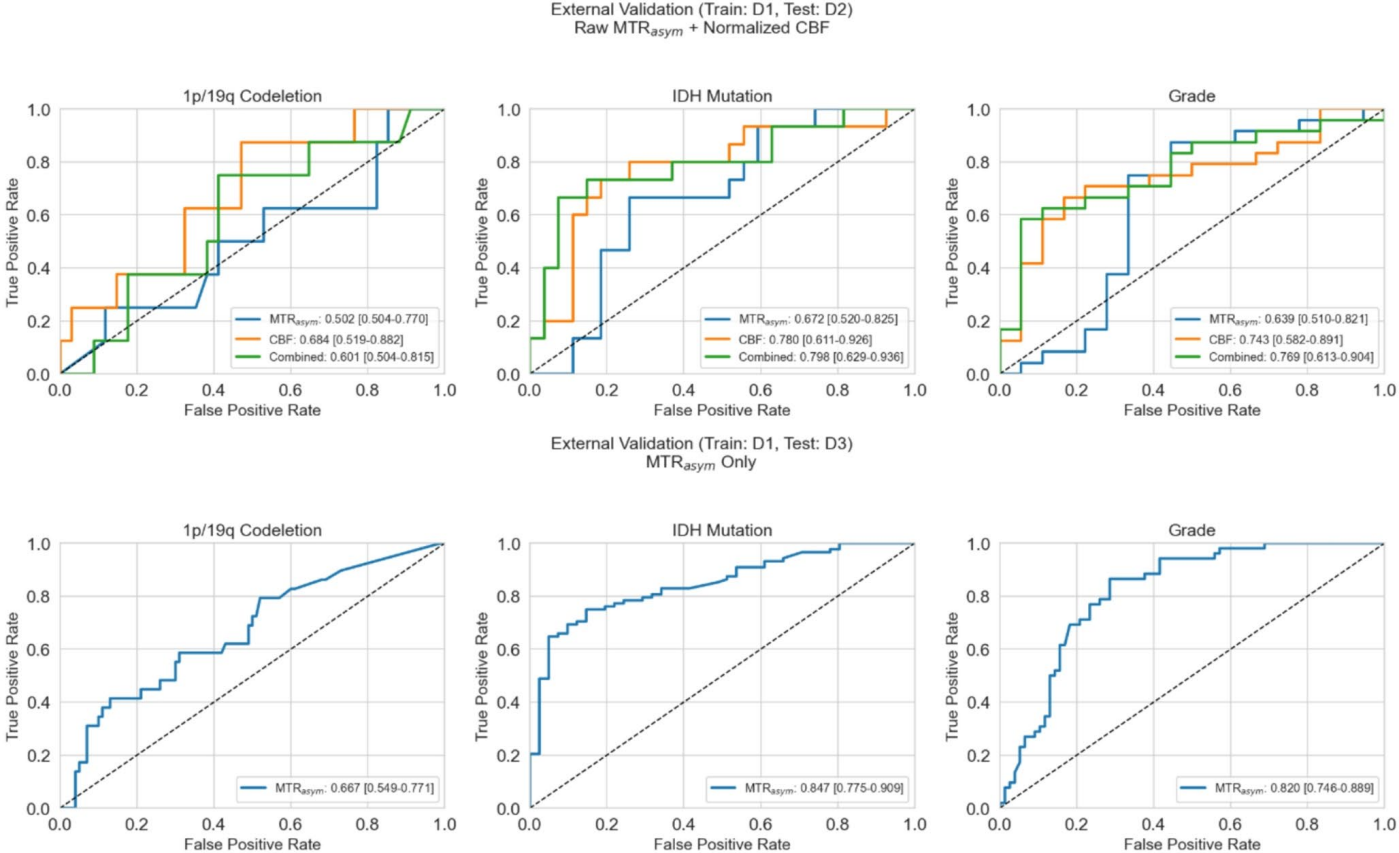
Area under the receiver operating characteristics curves (AUROC) for contralateral region of interest (ROI)-normalized cerebral blood flow (CBF), magnetization transfer ratio asymmetry (MTR_asym_) and/or combined (MTR_asym_+CBF) in predicting IDH status, 1p/19q codeletion status, and grade (high versus low) using random forest radiomics models on the D2 (top row) and D3 (bottom row) datasets

IDH and grade prediction using MTR_asym_ with the previously trained model demonstrated higher performance in D3 (*n* = 129; AUC = 0.82–0.85), compared to D2 (*n* = 42; AUC = 0.64–0.67) (see Fig. 7). The contralateral ROI-normalized CBF models demonstrated higher diagnostic performance when cross-validated on D2 compared to raw CBF models. Raw CBF results are shown in Fig. S5.

#### Combined dataset

**Fig. 8:**
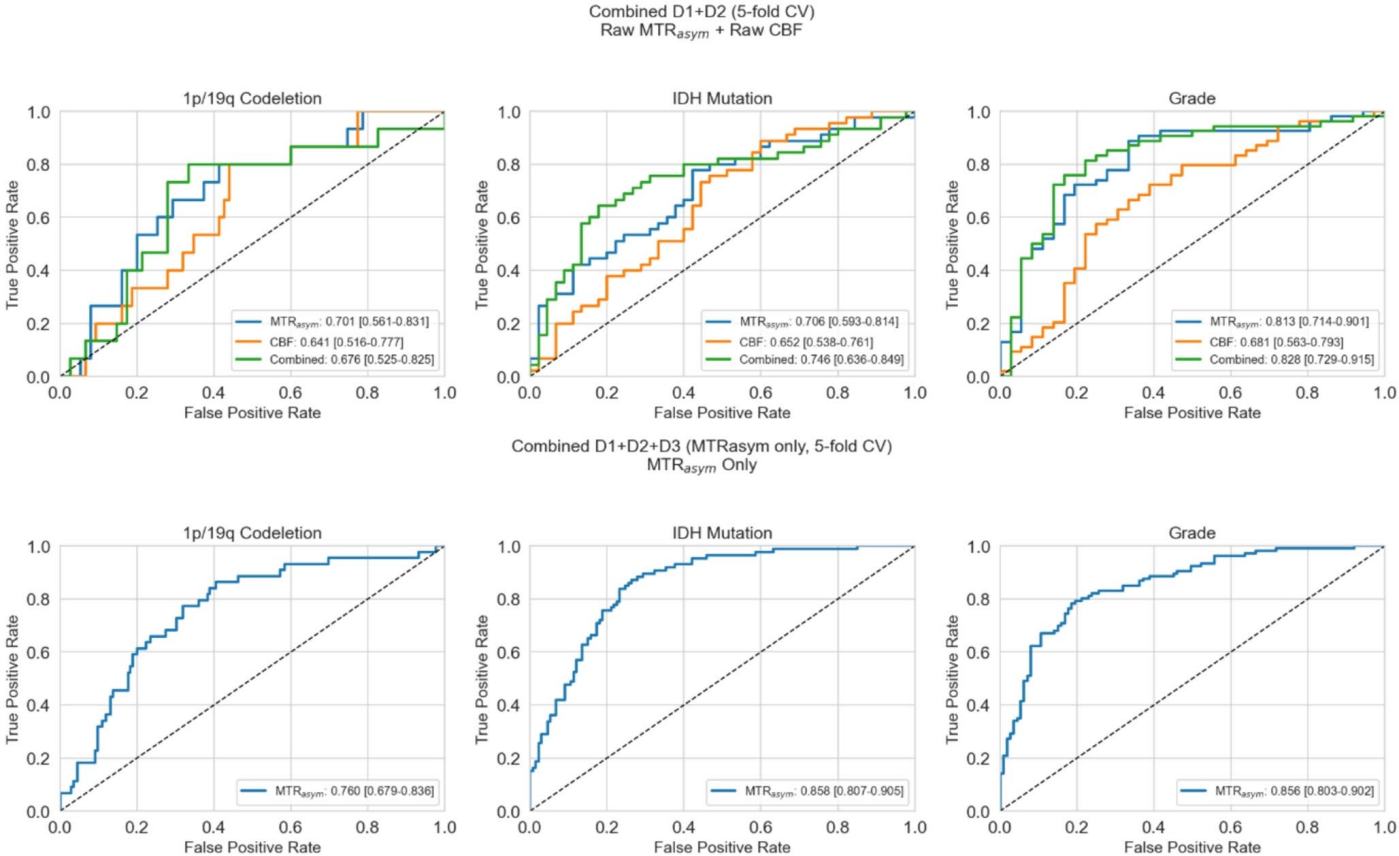
Area under the receiver operating characteristics curves (AUROC) for cerebral blood flow (CBF), magnetization transfer ratio asymmetry (MTR_asym_) and combined (MTR_asym_+CBF) in predicting IDH status, 1p/19q codeletion status, and grade (4 vs. 2/3) using random forest radiomics models in pooled datasets (D1 + D2 top row; D1 + D2+D3 bottom row) using 5-fold cross validation (CV)

When combining all three datasets (*n* = 219), MTR_asym_ achieved comparable classification performance for grade and IDH mutation compared to the D1-only radiomics analysis. In the two-site analysis combining the D1 and D2 datasets (*n* = 90), the combined MTR_asym_ + contralateral ROI-normalized CBF model demonstrated the highest performance for grade and IDH prediction; however, the improvement was not significant over the best performing single modality (*p* > 0.05). Figure 8 and S6 show contralateral ROI-normalized model results and raw model results, respectively.

## Discussion

This multi-center study demonstrates that combining APT-CEST and ASL-derived features improves the non-invasive prediction of IDH mutation status and WHO grade compared to single-modality approaches, supporting the integration of metabolic and perfusion imaging for their complementary tumor information. Furthermore, it shows that histogram-based radiomics can yield reproducible results for IDH and grade classification across datasets when coupled with appropriate normalization and harmonization strategies. However, cross-site performance did not fully match internal benchmarks.

Our analysis revealed significant differences in baseline tumor and/or contralateral ROI signal intensities across datasets, reflecting the well-documented sensitivity of both APT-CEST and ASL to acquisition parameters and scanner hardware [15]. Consequently, tumor-to-contralateral ratios showed no significant differences between datasets, suggesting that internal tissue normalization is a pragmatic approach for mitigating intra-site variability in MTR_asym_ and CBF-based features, similarly to amino-acid PET imaging [41].

Group comparisons revealed that MTR_asym_ and normalized CBF features demonstrated consistent and significant associations with glioma molecular subtypes, particularly when restricted to enhancing tumor regions. These findings are biologically plausible and corroborate prior literature on IDH [18, 19, 32, 34] and grade [32, 34] prediction. The MTR_asym_ signal at 3.5 ppm predominantly reflects mobile protein and peptide concentrations, which are elevated in more proliferative and metabolically active tumors [14–16]. IDH-wildtype gliomas and higher-grade tumors typically exhibit greater cellularity, increased protein synthesis, and altered pH—all factors that contribute to elevated MTR_asym_ signal [17, 19, 42]. The strong association with 1p/19q codeletion status may reflect the distinct metabolic phenotype of oligodendrogliomas. However, this finding should be interpreted cautiously, given the small number of codeleted tumors in all datasets. The significant CBF associations with IDH status and grade align with the known hypervascular phenotype of IDH-wildtype and high-grade tumors, particularly glioblastomas [22, 43, 44]. While IDH-wildtype glioblastomas are often hypervascular, considerable overlap exists with IDH-mutant astrocytoma grade 3 and 4 tumors. Importantly, normalized CBF showed comparable univariate diagnostic performance to MTR_asym_ for IDH mutation and 1p/19q codeletion prediction, suggesting that perfusion imaging provides complementary molecular information when appropriately normalized to account for inter-subject variability. Extension to include FLAIR hyperintense regions preserved the higher MTR_asym_ signal of glioblastomas relative to both oligodendrogliomas and astrocytomas, yet eliminated significant CBF between-group differences, suggesting that peritumoral infiltration introduces metabolic and vascular heterogeneity that diminishes could quantitative biomarker specificity in lower-grade lesions.

Beyond univariate associations, our radiomics models generally outperformed univariate features, indicating that the full radiomic feature set captures additional discriminative information beyond simple intensity statistics. The complementary contributions of MTR_asym_+CBF to model performance may reflect distinct but related aspects of tissue pathophysiology. For example, tumors may be highly cellular yet relatively hypoperfused, or moderately cellular yet hypervascular. The non-redundant nature of these signals means multiparametric integration can provide a more complete portrait of tumor biology. Prior clinical studies similarly showed added value when combining MTR_asym_ with perfusion measures for glioma grading [17, 42], and prior research also observed this synergistic performance in treatment-response assessment [45].

Cross-dataset validation revealed an intriguing pattern of complementary generalizability between modalities. MTR_asym_ models derived from D1 showed good generalization to D3 (Siemens scanner, matching our internal dataset) but poor performance on D2 (Philips scanner), while normalized CBF demonstrated the opposite pattern, outperforming MTR_asym_ on D2 for all classification tasks. This modality-specific vendor sensitivity may reflect differences in how CEST and ASL acquisitions are affected by scanner-dependent factors such as B0 and B1 inhomogeneities, labeling efficiency, and sequence implementation. Similarly to our internal radiomics model, multimodal classifiers demonstrated higher performance for both IDH mutation and grade classification tasks, however, these improvements did not reach statistical significance, likely due to the limited statistical power of the DeLong tests used in small samples (*n* = 42). Additionally, the observation that contralateral ROI-normalized ratios were comparable across sites despite large differences in absolute intensities suggests that the cross-site performance drop for MTR_asym_ in D2 may be primarily driven by systematic intensity shifts rather than more complex technical confounders.

A limitation of our current analysis is the difference and imbalance in tumor subtype distribution across sites, which may have contributed to some of the observed variability in reported population values. Another methodological consideration is that we did not have full control over the quantification process: we obtained pre-quantified data from site D2 while performing the quantification ourselves for D1; this inconsistency could further reduce reproducibility. Radiomic studies frequently suffer from spurious associations arising from high-dimensional data and oversampling - as well as poor reproducibility due to inconsistent ROI segmentation and lack of external reproducibility/validation [39, 46, 47]. This is exacerbated for ASL and APT-CEST, where sparse literature, technical heterogeneity, inconsistent ROI selection standards, and small datasets limit clinical translation [15]. We correct this by additionally evaluating the diagnostic performance on independent datasets. Given the strong univariate performance of simple intensity metrics, future work should investigate whether radiomic models offer true added value over parsimonious approaches using only a handful of features (e.g., mean, maximum, standard deviation), and whether appropriate reference tissue normalization could sufficiently address cross-vendor variability without requiring more complex harmonization strategies. Currently, z-score normalization is employed to equalize the results across datasets on top of normalization to the healthy tissue for ASL. Reproducibility of quantitative MRI remains a major impediment and several more advanced approaches should be used in further studies including equalizing sequences (for example using gammaStar framework) or harmonizing data using dedicated algorithms (such as NeuroComBat), which can account for batch effects and have been shown to improve between-site reproducibility in ASL [48].

In conclusion, both MTR_asym_ and CBF-based histogram features show promise for non-invasive prediction of glioma molecular characteristics, with contralateral ROI normalization potentially reducing inter-site variability. Cross-vendor validation reveals complementary patterns, with MTR_asym_ generalizing well to same-vendor sites and CBF outperforming MTR_asym_ on cross-vendor data. Combined MTR_asym_+CBF radiomics may offer modest advantages over single-modality approaches when both are available.

## Supplementary Information

Below is the link to the electronic supplementary material.

Supplementary Material 1 (DOCX 6.05 MB)

## Data Availability

The internal institutional dataset cannot be made publicly available due to patient privacy restrictions; however, descriptive statistics are provided in the Supplementary material. The external validation dataset from Filimonova et al. [1] is publicly accessible. The external validation dataset from Wu et al. [2] was obtained upon request and is subject to the sharing policies of the original authors.1. Filimonova E, Pashkov A, Borisov N, Kalinovsky A, Rzaev J. Utilizing the amide proton transfer technique to characterize diffuse gliomas based on the WHO 2021 classification of CNS tumors. The Neuroradiology Journal. 2024 Mar 28;37(4):490–9. https://doi.org/10.1177/197140092412426582. Wu M, Jiang T, Guo M, et al. Amide proton transfer-weighted imaging and derived radiomics in the classification of adult-type diffuse gliomas. European Radiology. 2023 Oct 19;34(5):2986–96. https://doi.org/10.1007/s00330-023-10343-6.
