# Supplementary material for: Amide proton transfer and arterial spin labeling for non-invasive molecular stratification of glioma: a multi-dataset imaging biomarker study
Source: Neuroradiology. 2026 Apr 23;68(5):1387–401. doi: 10.1007/s00234-026-04006-8 (PMC13216161; doi:10.1007/s00234-026-04006-8)
Supplement: Supplementary file 1 — Supplementary Material 1 (DOCX 6.05 MB) [file 234_2026_4006_MOESM1_ESM.docx]

# Supplementary material

List of all extracted first-order features:

1. Energy - The energy of voxel intensities​
2. Total Energy - The total energy of voxel intensities​
3. Entropy - Measure of randomness/uncertainty in intensity distribution​
4. Minimum - Minimum gray level intensity in the ROI
5. 10^th^ Percentile - The 10^th^ percentile of voxel intensities​
6. 90^th^ Percentile - The 90^th^ percentile of voxel intensities
7. Maximum - Maximum gray level intensity in the ROI​
8. Mean - Average gray level intensity​
9. Median - Middle value of the intensity distribution​
10. Interquartile Range - Difference between 75th and 25th percentiles​
11. Range - Difference between maximum and minimum values​
12. Mean Absolute Deviation (MAD) - Average absolute deviation from the mean
13. Robust Mean Absolute Deviation (rMAD) - MAD calculated using the 10^th^-90^th^ percentile range
14. Root Mean Squared (RMS) - Square root of the mean of squared intensities​
15. Standard Deviation - Measure of intensity variability​
16. Skewness - Measure of asymmetry of the distribution​
17. Kurtosis - Measure of the "tailedness" of the distribution (note: PyRadiomics calculates standard kurtosis, which is +3 compared to IBSI's excess kurtosis)​
18. Variance - Square of the standard deviation
19. Uniformity - Measure of homogeneity of intensity distribution

# Figures


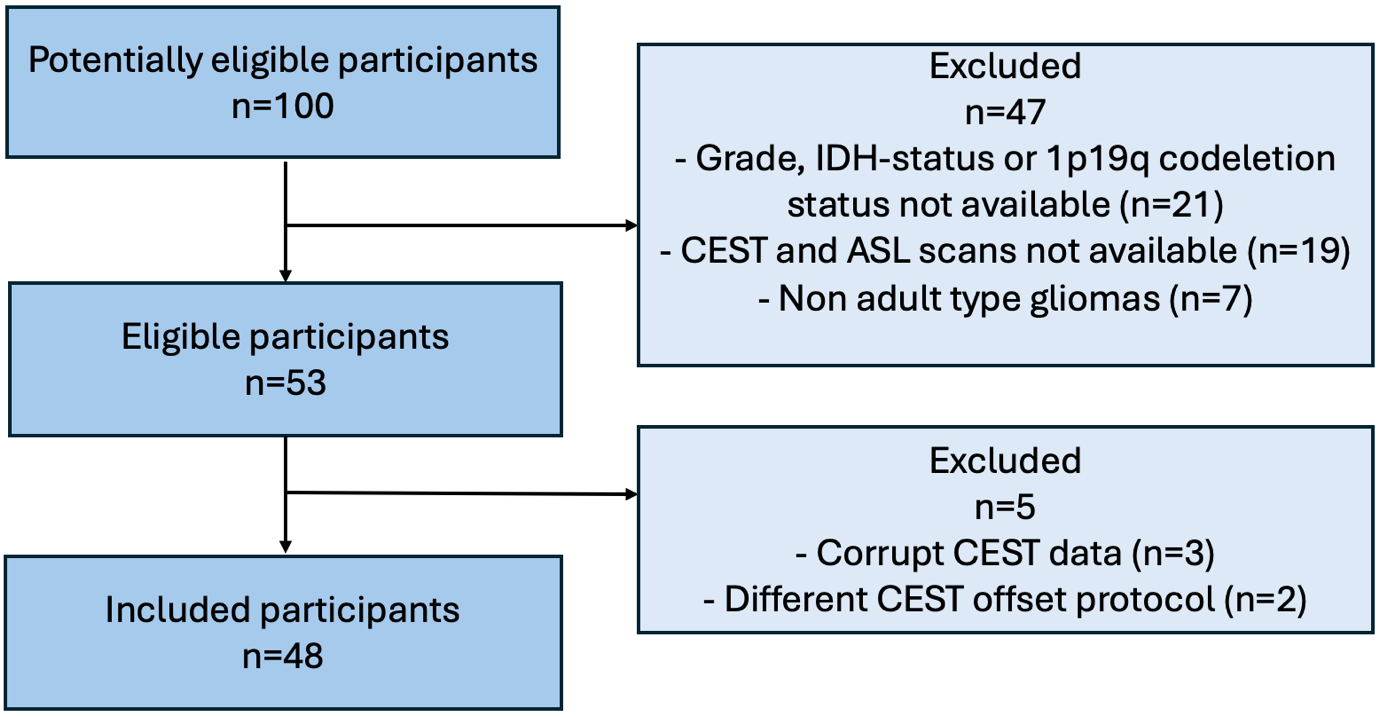


**Fig. S1**: Study inclusion flow diagram. CEST = chemical exchange saturation transfer; ASL = arterial spin labeling.


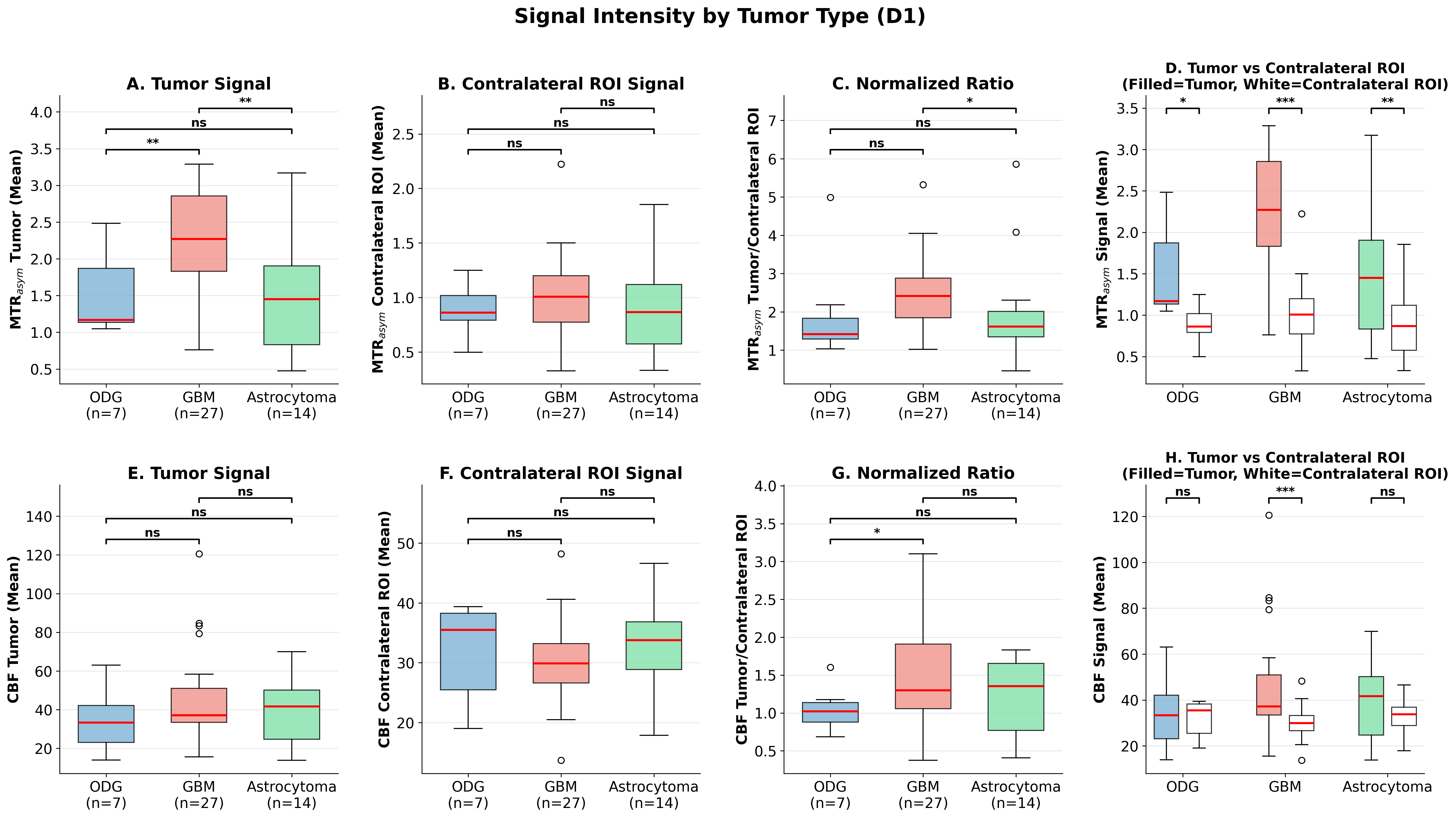


**Fig. S2a:** MTR_asym_(3.5ppm) and CBF signal intensity comparison across glioma subtypes in the D1 dataset. **(A)** Mean tumor MTR_asym_(3.5ppm) signal values stratified by histological subtype. **(B)** Mean contralateral region of interest (ROI) MTR_asym_(3.5ppm) signal showed no significant differences across tumor types. **(C)** Tumor-to-contralateral ROI MTR_asym_(3.5ppm) signal ratio did not differ significantly between groups. **(D)** Within-group comparison of tumor versus contralateral ROI MTR_asym_(3.5ppm) signal intensity. **(E)** Mean tumor CBF values stratified by histological subtype showed no significant differences. **(F)** Mean Contralateral ROI CBF signal showed no significant differences across tumor types. **(G)** Tumor-to-contralateral ROI CBF ratio did not differ significantly between groups. **(H)** Within-group comparison of tumor versus contralateral ROI CBF signal intensity.


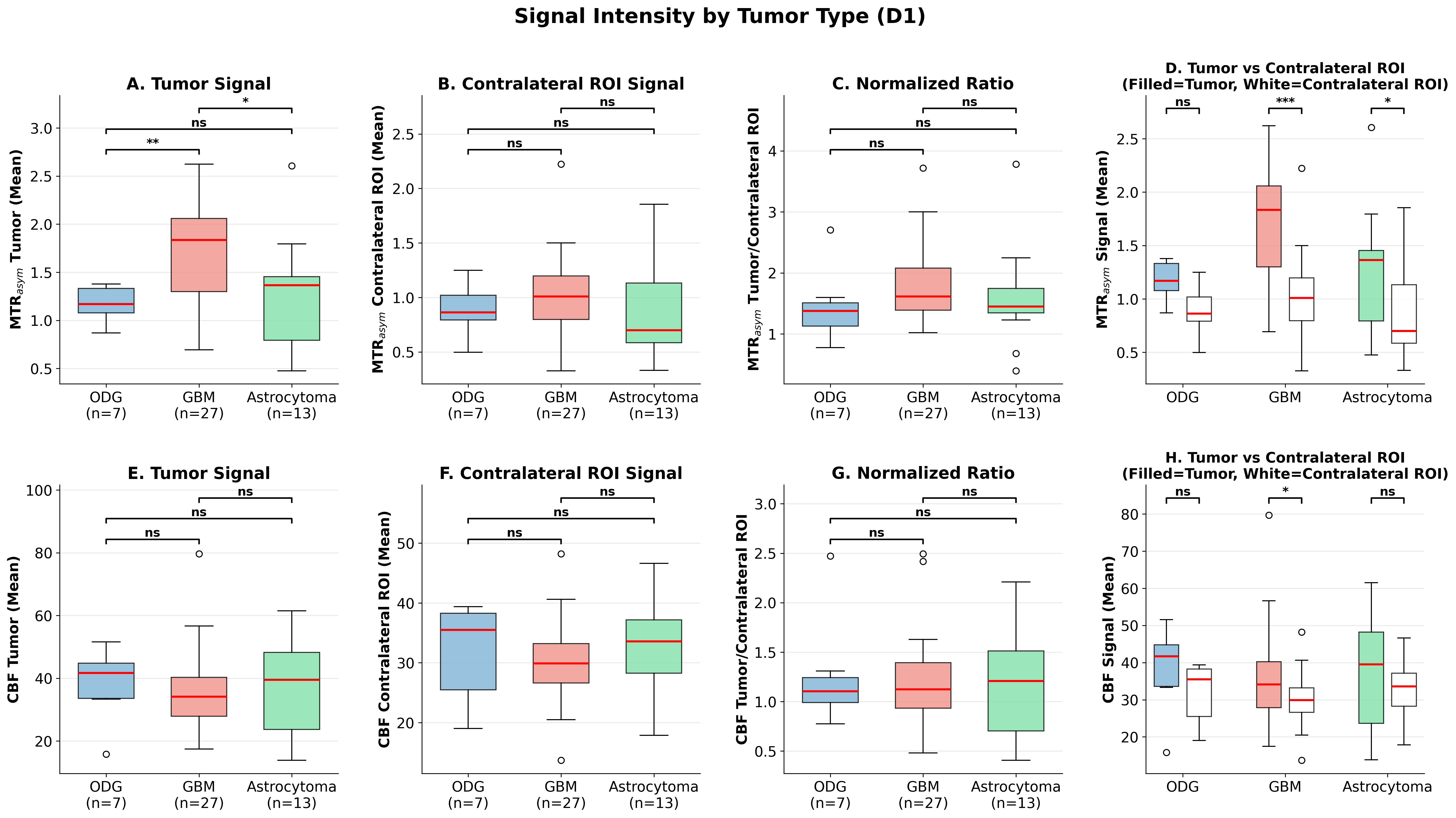


**Fig. S2b:** MTR_asym_(3.5ppm) and CBF signal intensity comparison across glioma subtypes in the D1 dataset using enhancing tumor + non-enhancing tumor/edema masks. **(A)** Mean tumor MTR_asym_(3.5ppm) signal values stratified by histological subtype. **(B)** Mean contralateral region of interest (ROI) MTR_asym_(3.5ppm) signal showed no significant differences across tumor types. **(C)** Tumor-to-contralateral ROI MTR_asym_(3.5ppm) signal ratio did not differ significantly between groups. **(D)** Within-group comparison of tumor versus contralateral ROI MTR_asym_(3.5ppm) signal intensity. **(E)** Mean tumor CBF values stratified by histological subtype showed no significant differences. **(F)** Mean Contralateral ROI CBF signal showed no significant differences across tumor types. **(G)** Tumor-to-contralateral ROI CBF ratio did not differ significantly between groups. **(H)** Within-group comparison of tumor versus contralateral ROI CBF signal intensity.


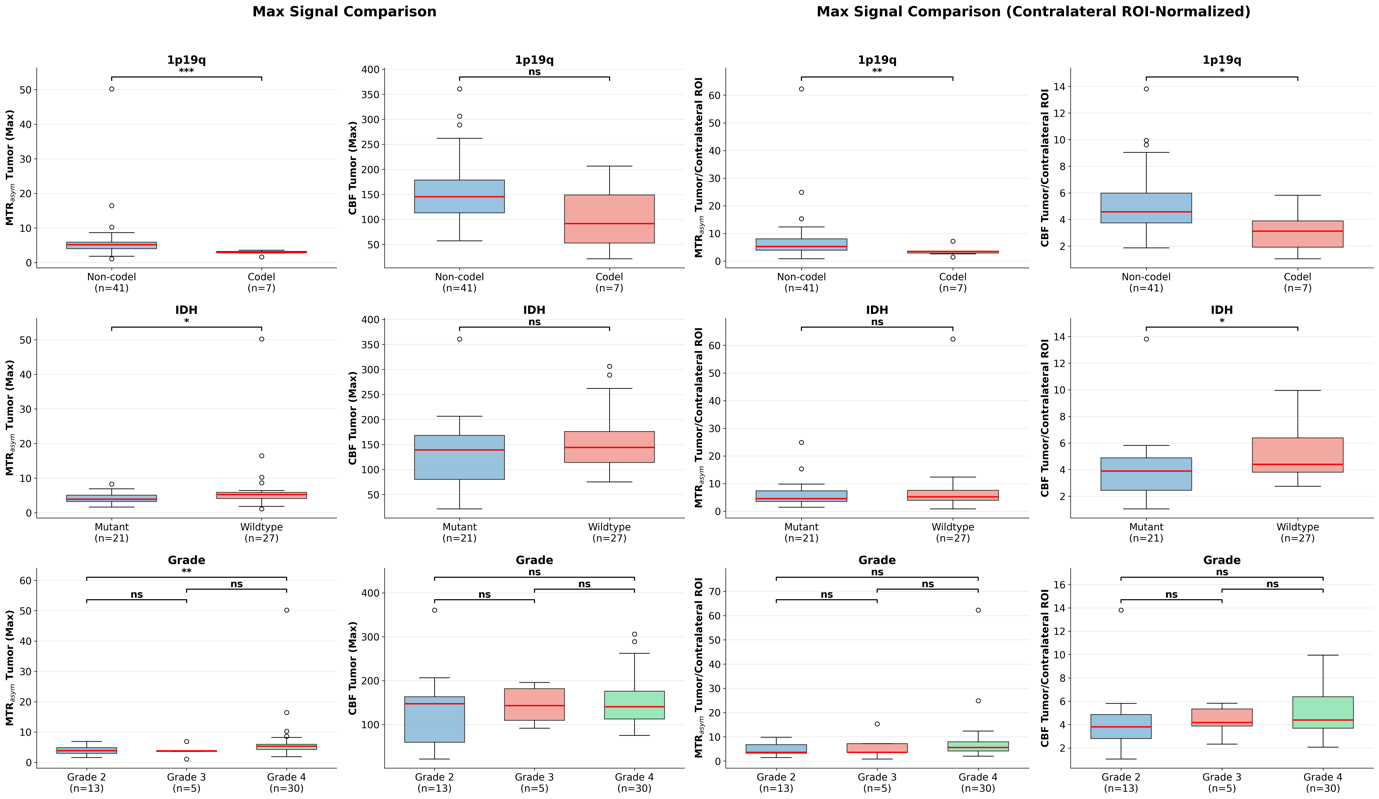


**Fig. S3:** Boxplots comparing raw (left 2x3) and contralateral region-of-interest (ROI)-normalized (right 2x3) maximum MTR_asym_ and cerebral blood flow (CBF) tumor values between groups for 1p/19q co-deletion status (row 1), IDH status (row 2) and grade (row 3) in dataset D1.


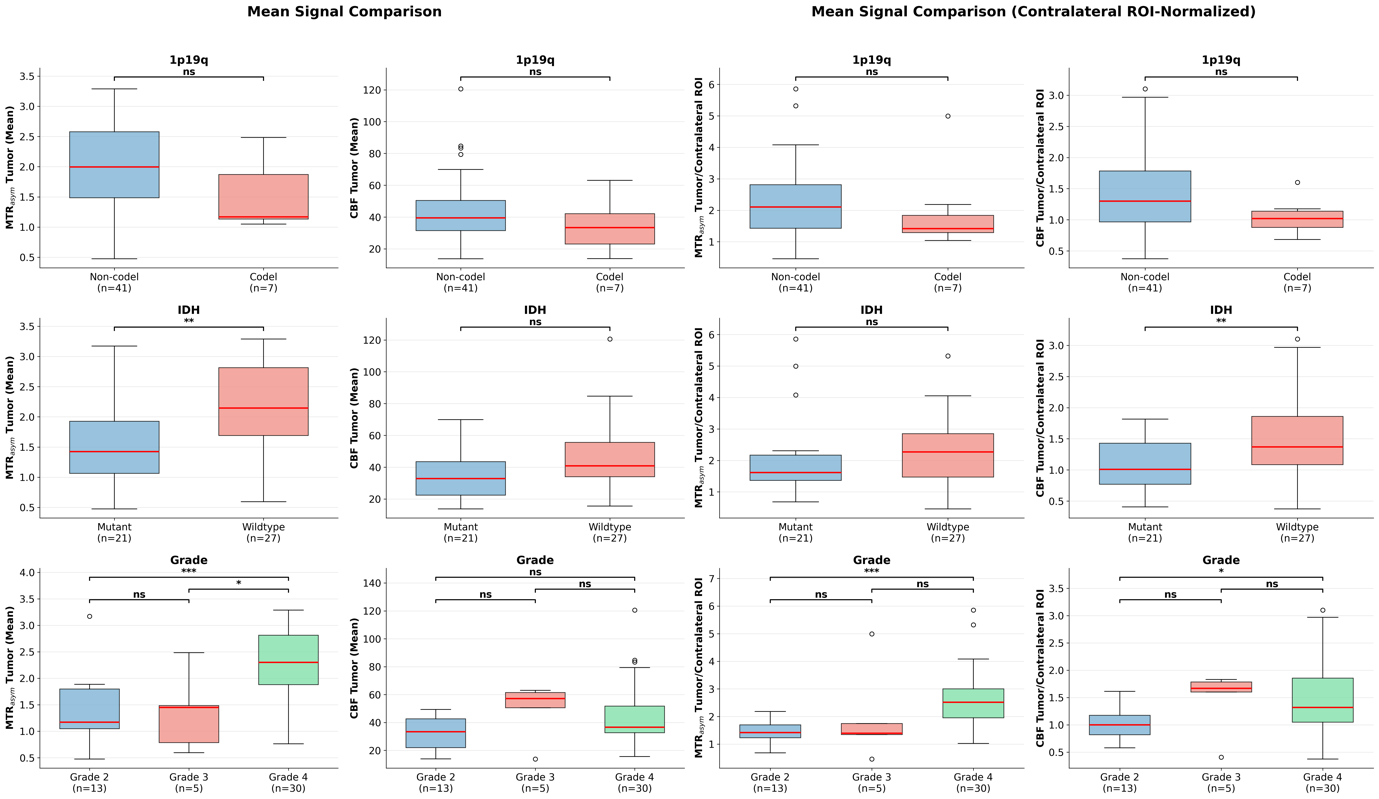


**Fig. S4:** Boxplots comparing raw (left 2x3) and contralateral region-of-interest (ROI)-normalized (right 2x3) mean MTR_asym_ and cerebral blood flow (CBF) tumor values between groups for 1p/19q co-deletion status (row 1), IDH status (row 2) and grade (row 3) in dataset D1.


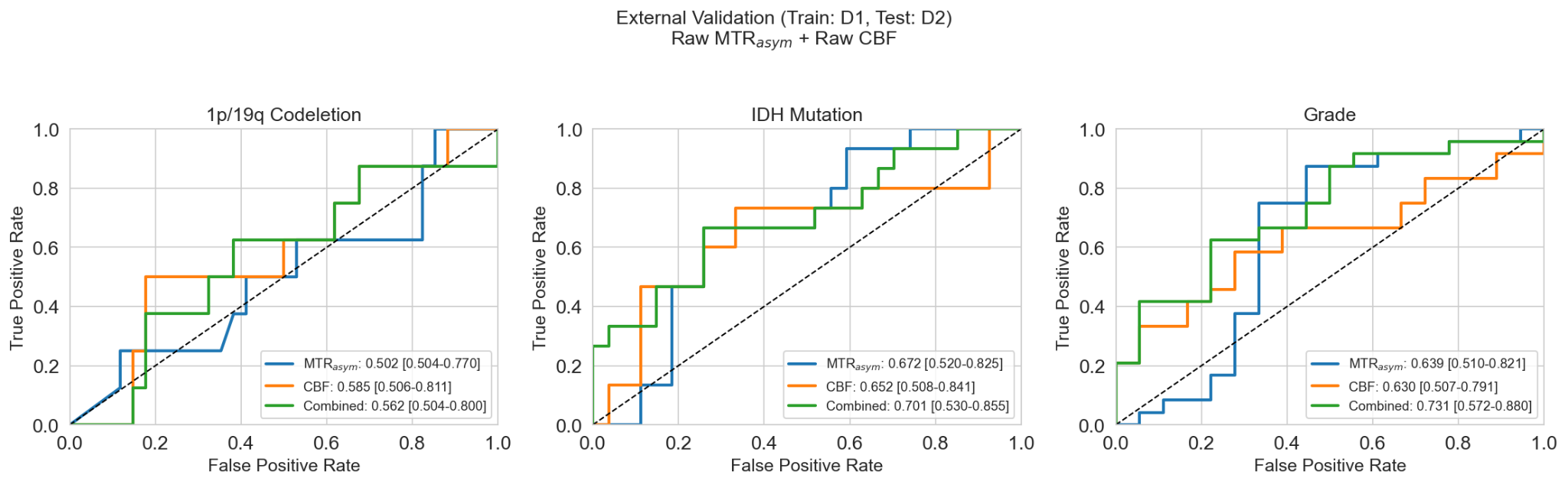


**Fig. S5:** Area under the receiver operating characteristics curves (AUROC) for cerebral blood flow (CBF), magnetization transfer ratio asymmetry (MTR_asym_) and combined (MTR_asym_+CBF) in predicting IDH status, 1p/19q codeletion status, and grade (high versus low) using random forest radiomics models trained on the D1 dataset and tested on the D2 dataset using Z-score feature normalisation/harmonization.


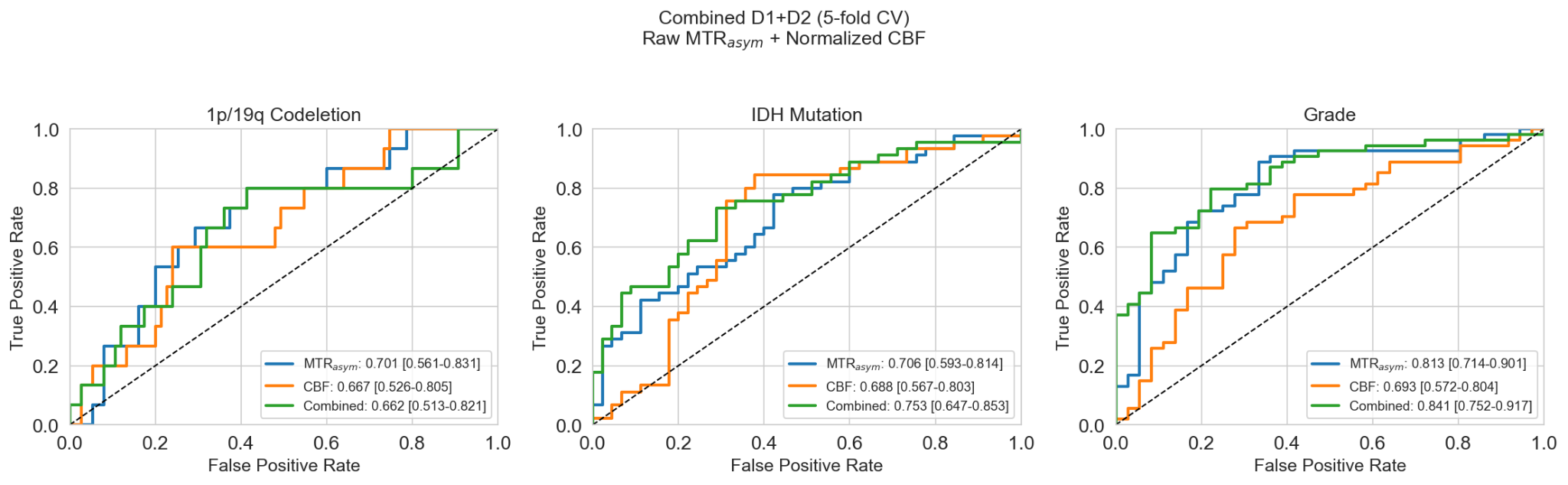


**Fig. S6:** Area under the receiver operating characteristics curves (AUROC) for cerebral blood flow (CBF), magnetization transfer ratio asymmetry (MTR_asym_) and combined (MTR_asym_+CBF) in predicting IDH status, 1p/19q codeletion status, and grade (4 versus 2/3) using random forest radiomics models in pooled datasets using raw MTR_asym_ and CBF with z-score normalisation on combined D1 and D2 datasets.

# Tables

| **Table S1: CEST imaging parameters** | | | | | | |
| --- | --- | --- | --- | --- | --- | --- |
| Dataset | Hardware | Saturation parameters | Offset frequencies | Readout | B0 correction | Voxel size |
| Netherlands | Vida Siemens 3T | Tsat=1.1s, tp=100ms, td=10ms, B1rms=2μT, DCsat=91% | ± 3ppm, ±3.5ppm, ±4ppm, -1560 ppm | 3D TSE SPACE-CEST | dual-echo GRE | 1.4x1.4x2.8 mm^3^ |
| Russia | Philips 3T | unknown | unknown | 3D TSE | unknown | 2x2x6 mm^3^ |
| China | Siemens Prisma 3T | Tsat=1s, tp=1s, B1rms=2.5μT, DCsat=100% | ± 3ppm, ±3.5ppm, ±4ppm, -1560 ppm | 3D TSE SPACE-CEST | dual-echo GRE | 2.8x2.8x2.8 mm^3^ |
| **Abbreviations**: 3D = three-dimensional, B0 = static magnetic field, B1 = radiofrequency field amplitude, B1rms = root mean square B1, DCsat = saturation duty cycle, GRE = Gradient Echo, ppm = parts per million, SPACE-CEST = Sampling Perfection with Application optimized Contrast using different flip angle Evolution - Chemical Exchange Saturation Transfer, td = delay between repeated RF saturation pulses, tp = saturation pulse length, Tsat = saturation time, TSE = Turbo Spin Echo | | | | | | |

| **Table S2: ASL imaging parameters** | | | | | | | | |
| --- | --- | --- | --- | --- | --- | --- | --- | --- |
| Dataset | Hardware | Sequence | Background suppression | TR/TE (ms) | Labeling duration (ms) | PLD times | Readout | Voxel size (mm^3^) |
| Netherlands | Vida Siemens 3T | pCASL | Yes | 5000/22.1 | 1800 | 1600  2400  3200  3600  3800  4000  4200  4400 | 3D GRASE | 1.8x1.8x4 |
| Russia | Philips 3T | pCASL | Unknown | 4300/11.6 | 1800 | Unknown | 3D | 3.75x3.75x6 |
| **Abbreviations**: 3D = three-dimensional, GRASE = Gradient and Spin Echo, pCASL = pseudo Continuous Arterial Spin Labeling, PLD = Post-Labeling Delay, TE = Echo Time, TR = Repetition Time | | | | | | | | |

| **Table S3: Absolute and normalized histogram feature values for different tumor molecular subtypes and grades** | | | | | | | | | |
| --- | --- | --- | --- | --- | --- | --- | --- | --- | --- |
| **Comparison** | **Modality** | **Group** | **N** | **Mean (abs)** | **90th Percentile (abs)** | **Maximum (abs)** | **Mean (norm)** | **90th Percentile (norm)** | **Maximum (norm)** |
| 1p/19q codeletion | APT | Non-codeleted | 41 | 2.01 ± 0.75 | 2.74 ± 0.87 | 6.48 ± 7.32 | 2.90 ± 2.91 | 1.81 ± 0.82 | 0.85 ± 0.59 |
| 1p/19q codeletion | APT | 1p/19q codeleted | 7 | 1.53 ± 0.51 | 2.06 ± 0.55 | 2.88 ± 0.58 | 3.18 ± 3.47 | 1.45 ± 0.83 | 0.42 ± 0.24 |
| 1p/19q codeletion | CBF | Non-codeleted | 41 | 43.27 ± 21.21 | 74.79 ± 29.98 | 154.82 ± 63.07 | 1.27 ± 0.65 | 1.10 ± 0.45 | 1.02 ± 0.49 |
| 1p/19q codeletion | CBF | 1p/19q codeleted | 7 | 34.39 ± 15.33 | 53.75 ± 19.84 | 103.26 ± 61.86 | 0.96 ± 0.31 | 0.80 ± 0.38 | 0.48 ± 0.42 |
| IDH | APT | IDH mutant | 21 | 1.57 ± 0.69 | 2.16 ± 0.71 | 4.32 ± 1.66 | 3.24 ± 3.28 | 1.65 ± 0.95 | 0.80 ± 0.77 |
| IDH | APT | IDH wildtype | 27 | 2.17 ± 0.67 | 2.93 ± 0.82 | 6.93 ± 8.46 | 2.75 ± 2.80 | 1.83 ± 0.73 | 0.78 ± 0.41 |
| IDH | CBF | IDH mutant | 21 | 35.11 ± 15.55 | 59.42 ± 24.73 | 133.96 ± 75.77 | 1.00 ± 0.36 | 0.86 ± 0.31 | 0.85 ± 0.63 |
| IDH | CBF | IDH wildtype | 27 | 46.09 ± 22.25 | 79.10 ± 29.94 | 155.31 ± 56.96 | 1.35 ± 0.70 | 1.17 ± 0.48 | 0.99 ± 0.42 |
| Grade | APT | Grade 2 | 13 | 1.40 ± 0.65 | 1.98 ± 0.67 | 3.97 ± 1.37 | 1.91 ± 0.92 | 1.25 ± 0.32 | 0.56 ± 0.32 |
| Grade | APT | Grade 3 | 5 | 1.36 ± 0.66 | 1.88 ± 0.69 | 3.86 ± 1.83 | 3.42 ± 4.03 | 1.58 ± 1.01 | 0.95 ± 0.56 |
| Grade | APT | Grade 4 | 30 | 2.27 ± 0.57 | 3.06 ± 0.69 | 7.16 ± 8.40 | 3.30 ± 3.27 | 2.01 ± 0.83 | 0.86 ± 0.63 |
| Grade | CBF | Grade 2 | 13 | 32.67 ± 11.66 | 56.56 ± 20.01 | 137.35 ± 83.80 | 1.03 ± 0.40 | 0.90 ± 0.33 | 0.90 ± 0.68 |
| Grade | CBF | Grade 3 | 5 | 49.18 ± 18.22 | 74.27 ± 24.45 | 144.26 ± 40.04 | 1.02 ± 0.29 | 0.88 ± 0.27 | 0.99 ± 0.50 |
| Grade | CBF | Grade 4 | 30 | 44.80 ± 22.74 | 77.86 ± 31.57 | 152.12 ± 59.01 | 1.34 ± 0.70 | 1.15 ± 0.49 | 0.95 ± 0.42 |
| Abbreviations: 1p/19q = Chromosome 1p and chromosome 19q, abs = absolute, APT = Amide Proton Transfer, CBF = Cerebral Blood Flow, IDH = Isocitrate dehydrogenase, N = Number of patients, norm = normalized by contralateral normal appearing white matter | | | | | | | | | |

| **Table s4: Univariate features versus radiomics model performance comparison** | | | | |
| --- | --- | --- | --- | --- |
| **Classification Task** | **Modality** | **Best Univariate Feature** | **Univariate AUC** | **Random Forest AUC** |
| **1p/19q Codeletion** | MTR_asym_ | Max (raw) | **0.944 (0.865–1.000)** | 0.913 (0.888–0.937) |
|  | CBF | 90^th^ %ile (norm) | 0.753 (0.591–0.892) | **0.805 (0.761–0.849)** |
|  | Combined | Max (raw / norm) | 0.847 (0.665–0.978) | **0.900 (0.867–0.933)** |
| **IDH Status** | MTR_asym_ | 90^th^ %ile (raw) | 0.761 (0.614–0.889) | **0.806 (0.769–0.844)** |
|  | CBF | 90^th^ %ile (norm) | **0.769 (0.620–0.902)** | 0.743 (0.701–0.785) |
|  | Combined | 90^th^ %ile (raw / raw) | 0.806 (0.657–0.923) | **0.854 (0.821–0.887)** |
| **Grade (High vs Low)** | MTR_asym_ | 90^th^ %ile (raw) | 0.865 (0.750–0.962) | **0.888 (0.857–0.919)** |
|  | CBF | 90^th^ %ile (norm) | 0.715 (0.555–0.865) | **0.787 (0.754–0.821)** |
|  | Combined | 90^th^ %ile (raw / norm) | 0.876 (0.767–0.966) | **0.913 (0.888–0.937)** |
| **Abbreviations**: 1p/19q = Chromosome 1p and chromosome 19q, %ile = percentile, AUC = Area Under the Curve, CBF = Cerebral Blood Flow, IDH = Isocitrate dehydrogenase, Max = Maximum, MTR_asym_ = Magnetization Transfer Ratio asymmetry, vs = versus | | | | |
